# Nutrient Separation Systems: Current Progress and Future Opportunities

**DOI:** 10.1021/acsestengg.5c00743

**Published:** 2026-01-20

**Authors:** Hyuck Joo Choi, Mohammed Tahmid, Luisa Barrera, Christian E. Alvarez-Pugliese, Danae A. Chipoco Haro, Dylan J. Weber, Wilfredo J. Cardona Velez, Bengu Mete, Dayana Donneys-Victoria, Zhengwen Zhang, Victor K. Lim, Olatunde D. Akanbi, Jacob D. Hostert, Archer Montgomery, Divya Ganesan, Erika I. Barcelos, Jie Xu, Joseph K. Scott, Gerardine G. Botte, Kayleigh Millerick, Chris Yuan, Julie N. Renner, Roger H. French, Marta C. Hatzell

**Affiliations:** † School of Chemical and Biomolecular Engineering, 1372 Georgia Institute of Technology, Atlanta, Georgia 30309, United States; ‡ George W. Woodruff School of Mechanical Engineering, 115724 Georgia Institute of Technology, Atlanta, Georgia 30309, United States; § Chemical & Electrochemical Technology & Innovation Lab, Institute for Sustainability and Circular Economy, Dept. of Chemical Engineering, 6177 Texas Tech University, Lubbock, Texas 79409, United States; ∥ School of Materials Science & Engineering, Georgia Institute of Technology, Atlanta, Georgia 30332, United States; ⊥ Dept. of Mechanical & Aerospace Engineering, 2546 Case Western Reserve University, Cleveland, Ohio 44106, United States; # Intelligent Sustainable Technologies Division, 457006 Georgia Institute of Technology, Atlanta, Georgia 30318, United States; ∇ Dept. of Materials Science & Engineering, 2546 Case Western Reserve University, Cleveland, Ohio 44106, United States; ○ Dept. of Chemical Engineering, 2546 Case Western Reserve University, Cleveland, Ohio 44106, United States; ◆ Dept. of Civil, Environmental & Construction Engineering, 6177 Texas Tech University, Lubbock, Texas 79409, United States

**Keywords:** nutrient recovery, nutrient-rich wastewater, nitrogen and phosphorus, ammonia recovery

## Abstract

As energy, environmental, supply chain, and economic risks escalate in today’s linear fertilizer manufacturing processes, there has been growing interest in developing technologies that enable a circular nitrogen-based fertilizer economy. Achieving this goal requires significant advancements in wastewater treatment, with a specific focus on the design of technologies and complete systems that can capture and recycle waste nutrients into usable fertilizers. Every year, millions of tons of nitrogen and phosphorus remain untapped in global municipal and industrial wastewater, presenting a significant opportunity for fertilizer utilization. Herein, we explore current and future opportunities for nutrient recovery systems to provide recycled fertilizers for agricultural use. We first quantify recoverable nutrient wastewater sources, examine current nutrient management processes (e.g., nitrification–denitrification, EBPR), and highlight the performance and limitations of current nutrient management processes. We also review the current commercialization landscape for nutrient recovery systems and detail efforts made in advancing full-scale deployments. Finally, we review emerging electrified technologies and compare nutrient recovery technologies in terms of technology readiness, scalability, optimal feedstock, and environmental trade-offs, pairing them with optimal wastewater feed streams. A gap analysis is also conducted to guide future research and development efforts in nutrient recovery.

## Graphical abstract

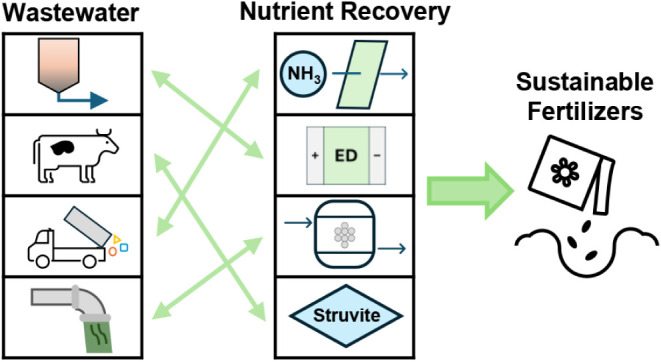

## 1 Introduction

The effective management of nitrogen and phosphorus cycles has become critical as concentrations of nitrogen (NO_*x*_, NH_*x*_, and organic N) and phosphorus (organic P and PO_4_^3–^) rise in aquatic and atmospheric environments. Excess nutrients in surface waters cause eutrophication and harmful algal blooms, which deplete dissolved oxygen, produce toxins (microcystins), and devastate local ecosystems, thereby impacting public health.^1^ Annually, 16.6 million tons of nitrogen and 3 million tons of phosphorus are estimated to be untapped in global wastewater.^2^ Excess atmospheric emissions of NH_3_ and NO_*x*_ also form fine aerosol nitrates (NH_4_NO_3_), negatively affecting public health.^3^ For those reasons, excess nitrogen waste management was identified as one of the 14 grand challenges of the National Academy of Engineering (NAE) and was mentioned in two of the five grand challenges described in the NAE report on the future of environmental engineering.^4–6^

Municipal wastewater treatment plants (WWTPs), industrial installations, and agricultural runoff generate the bulk of nitrogen- and phosphorus-rich wastewater. Unlike industrial settings, where wastewater is typically managed on-site, human waste in cities is collected and treated through centralized systems such as municipal WWTPs. These municipal systems rely on biological nitrification–denitrification for nitrogen removal using autotrophic bacteria or heterotrophic bacteria and enhanced biological phosphorus removal (EBPR) for phosphorus removal using polyphosphate-accumulating organisms (PAOs).^7–9^ Certain PAOs used in EBPR could denitrify organisms (denitrifying polyphosphate-accumulating organisms), producing nitrous oxide (N_2_O) as a byproduct. This highly limits EBPR’s potential for nitrogen recovery despite being an efficient method of phosphorus recovery.^10^

Agricultural sites also contribute substantially to nutrient pollution through agricultural runoff, animal feeding operations (AFOs), and concentrated animal feeding operations (CAFOs) lagoons. Direct land application of lagoon wastewater, known as spray culture, remains the most common commercial application for recovering nutrients in CAFO lagoons in the U.S.^11^ However, volatilization of nitrogen nutrients as ammonia gas significantly reduces the amount of applicable nutrients by 50–75%.^12,13^

Due to the spatiotemporal variability of wastewater composition, U.S. nutrient discharge levels are governed by facility-specific National Pollutant Discharge Elimination System (NPDES) permits rather than a single federal limit. At the federal level, under the Clean Water Act, the U.S. Environmental Protection Agency (EPA) provides Effluent Limitations Guidelines (ELG) as technology-based baselines, which states then translate into site-specific NPDES nutrient limits.^14^ Reported NH_3_–N limits through ELG range from 8 to 100 mg/L, NO_3_^–^–N up to 30 mg/L, and total phosphorus of 0.5–100 mg/L.^14^

Despite effective removal, conventional WWTPs and agricultural practices follow a “waste-and-release” model rather than a true resource recovery process. To close the nutrient loop, researchers have developed a spectrum of chemical, physical, and biological separation methods.^15,16^ Historically, chemical precipitation (struvite and calcium phosphate), adsorption, selective ion exchange, breakpoint chlorination, and ammonia stripping are the most established and widely used separation technologies.^17^ Chemical precipitation recovers both nitrogen and phosphorus by adding magnesium or calcium to precipitate struvite (MgNH_4_PO_4_·6H_2_O) or calcium phosphates, which can be used directly as fertilizers.^18^ Adsorption and ion exchange processes introduce materials that selectively adsorb or bind and desorb or release nitrogen or phosphorus nutrients, offering a simple process with minimal waste.^19,20^ Ammonia stripping exploits the thermodynamic equilibrium between ammonium (NH_4_^+^) and ammonia (NH_3_) and is a popular recovery method for stripping. This technology achieves a high concentration factor that enables the production of streams with nutrient concentrations needed for fertilizer use. Emerging technologies include, but are not limited to, biologically assisted precipitation, reverse osmosis, forward osmosis, and electrodialysis, which have recently been studied for nutrient recovery purposes at the lab scale or pilot scale.^16,21^ These technologies present advantages in energy consumption, product throughput, or chemical input compared to conventional nutrient recovery systems but suffer from membrane fouling, lack of selectivity, and lack of full-scale or pilot-scale implementation for nutrient recovery purposes. This diverse set of nutrient recovery technologies and variability across wastewater streams requires meticulous pairing of recovery technologies with appropriate wastewater streams.

This review aims to introduce and couple nutrient recovery technologies with appropriate wastewater streams to maximize nutrient recovery and stable operation. In the sections that follow, we first establish the magnitude of untapped nutrient resources by quantifying recoverable nitrogen and phosphorus in municipal and agricultural wastewater. We then critically examine existing nutrient management processes, highlighting their operational performance, recovery efficiencies, and key limitations. Building on this foundation, we survey full-scale commercial deployments and leading industrial players to illustrate how these technologies have been implemented at a scale. Next, we compare and evaluate these established methods with emerging separation approaches in terms of recovery mechanisms, energy consumption, and cost. Finally, we integrate these insights into a unified framework that assesses technological readiness, scalability, optimal feedstock, and environmental trade-offs, thereby identifying critical gaps and guiding future research toward truly circular, resource-efficient nutrient recovery solutions.

## 2 Current Landscape of Nitrogen and Phosphorus Nutrient Availability

The U.S. demand for nitrogen and phosphorus fertilizers has risen sharply over the past six decades: nitrogen consumption increased from 2.5 million to 11.8 million tons, and phosphorus consumption increased from 1.0 million to 1.7 million tons.^22–24^ Specifically, the 2015 U.S. N–P–K consumption ratio of 59–19–22 underscores the predominant demand for nitrogen fertilizers. Simultaneously, demand for high nitrogen concentration fertilizers (>30 N wt %) has increased. For example, urea (46 N wt %) and nitrogen solution (32 N wt %) represented 25% and 43%, respectively, of the annual nitrogen consumption in 2015.^22,25^ Such demand for nitrogen nutrients motivated the use of nitrogen–phosphorus fertilizers (diammonium phosphate, monoammonium phosphate, etc.), which represent 91% of annual phosphorus fertilizer consumption. The costs of these fertilizers also increased significantly. Urea and nitrogen solution prices increased from $101 and $74 per ton in 1966 to $737 and $526 per ton of fertilizer in 2025, while nitrogen phosphates increased from $120 to $960 per ton during the same period. These trends—high nutrient concentration requirements, escalating costs, and price volatility—have driven interest in recovering nitrogen and phosphorus from waste streams. To guide technology selection, we first identify nutrient-rich point sources and survey nutrient concentrations and volumes. Next, we examine how advanced spatiotemporal approaches help assess dynamic changes in nutrient availability.

### 2.1 Nitrogen and Phosphorus Availability in Wastewater

To accurately evaluate the feasibility of nutrient recovery systems, we compile nutrient concentrations and key characteristics in major agricultural wastewater sources such as fertilizer production effluent,^27–38^ landfill leachate,^39–49^ concentrated animal feeding operations (CAFOs),^50–59^ agricultural runoff,^60–69^ and municipal wastewater treatment plant influent^70–73^ (Figure 1). Values were extracted from wastewater monitoring reports, academic papers, and government reports spanning the period 1995–2021. Chemical oxygen demand (COD) and biological oxygen demand (BOD) characterize the load of organics in different waste streams. COD quantifies the dissolved oxygen required to chemically treat any organic contaminants present in the waste stream, with BOD representing the subsection of organic contaminants requiring biochemical treatment.^74^ Wide ranges of concentrations and oxygen demand values were reported due to significant variability across local environments. The average pH of each waste stream was neutral in the range of 6.52–7.65.

**Figure 1:**
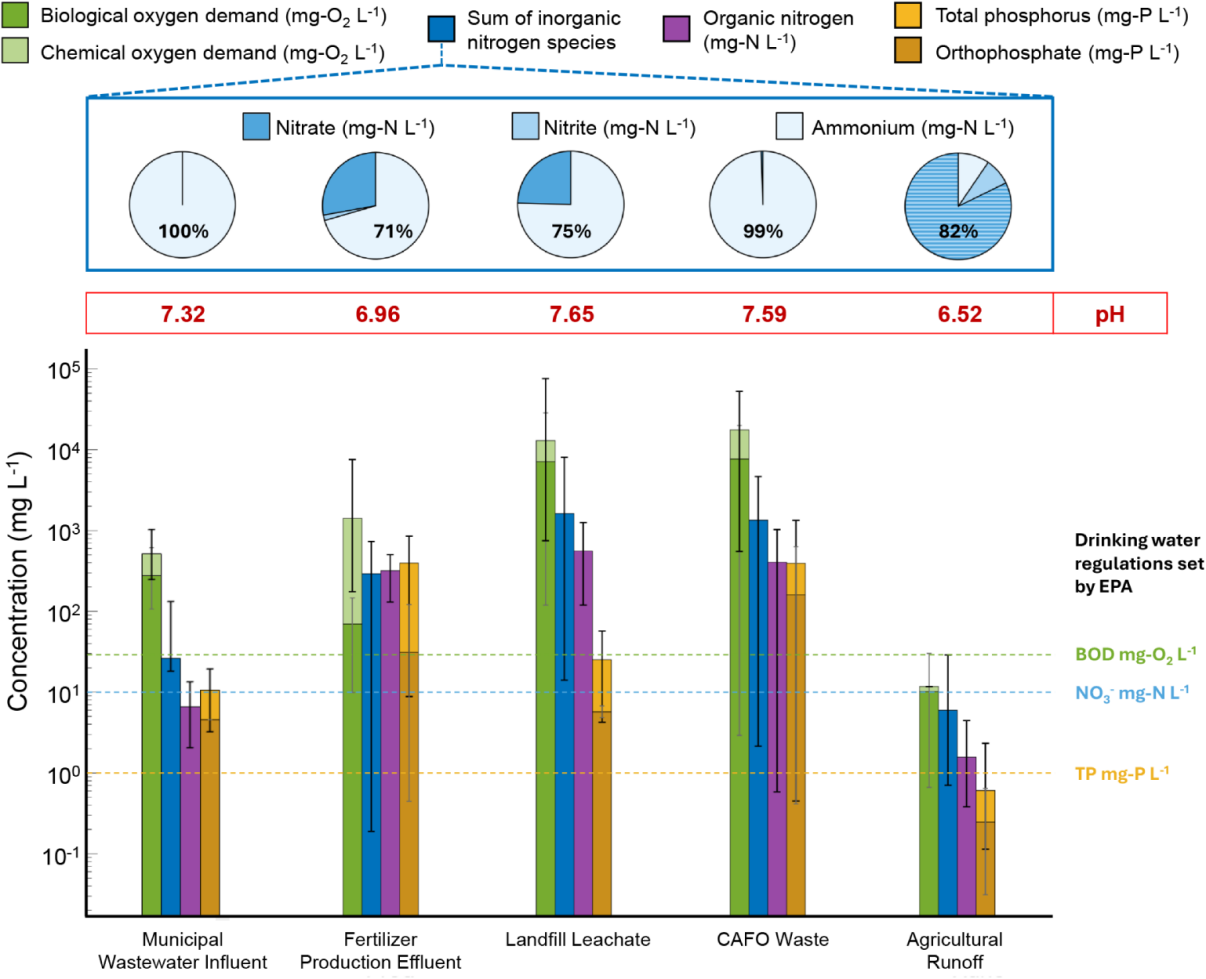
Average concentrations of biological and chemical oxygen demand (mg-O_2_/L), inorganic nitrogen species (mg-N/L), organic nitrogen species (mg-N/L), and total phosphorus and orthophosphate (mg-P/L) in different waste streams. Bars indicate the maximum and minimum values reported for all of the waste streams considered. Dashed lines indicate the maximum concentrations in drinking water allowed by the U.S. Environmental Protection Agency: green for BOD (30 mg-O_2_/L), blue for NO_3_^–^ (10 mg-N/L), and yellow for total phosphorus (1 mg-P/L). Corresponding pH values for each wastewater are shown above each bar.^26^ Reproduced from ref 25. Copyright 2021 American Chemical Society.

Fertilizer production effluent, landfill leachate, CAFO lagoon surface water, and municipal wastewater influent exhibit ammonia-dominant inorganic nitrogen concentrations of roughly 200, 1,200, 1,300, and 26 mg-N/L, respectively. Agricultural runoff contains higher concentrations of nitrate than ammonia, with concentrations of 5 mg-N/L. Total phosphorus concentrations are generally at similar or lower magnitudes than those of inorganic nitrogen concentrations. This allows technologies to recover both nitrogen and phosphorus from the same wastewater source.

The amount of nutrients in agricultural wastewater from Figure 1 (excluding agricultural runoff as it is a nonpoint source and is thus difficult to quantify) was equivalent to 28% of the annual ammonia and 23% of the annual phosphorus consumption for fertilizers in the United States in 2024 (Table 1).^22–24^ Municipal wastewater influent offers the largest volumetric flow and amount of available nutrients, but the dilute nutrient concentrations necessitate highly selective separation, followed by concentration processes. In contrast, landfill leachate demonstrates moderate discharge volumes and high concentrations of NH_4_^+^–N at ∼1500 mg-N/L. CAFO waste streams also have high ammonium concentrations of ∼1500 mg-N/L but with less than 1% of the landfill leachate volume. Thus, landfill leachate and CAFO waste streams would be attractive targets for recovery technologies optimized at mid to high nitrogen concentrations. In addition, CAFO waste also contains high phosphorus concentrations of ∼400 mg of P/L, making it appealing for phosphorus recovery technologies such as struvite precipitation. Finally, the BOD, COD, and pH values are also critical factors to consider when evaluating technology matching. Excess organics could inhibit performance through membrane fouling, reduce struvite crystallization kinetics, and affect solution buffer capacities.^78,79^

**Table 1:** Estimated Annual Wastewater Volumes in the U.S. for 2020 and the Associated Potential Nitrogen and Phosphorus Recovery^a^ ^75–77^

Wastewater Source	Annual WW Volume (million m^3^)	Total N (million ton-N)	Total P (million ton-P)
Municipal Wastewater Influent^75^	47240.0	3.0	0.3
Landfill Leachate^76^	874.4	1.9	0.02
CAFO Waste^76^	6.8	0.02	0.003
Fertilizer Production^76^	109.4	0.07	0.04

a Recovery estimates of agricultural wastewater are based on the average nutrient concentrations shown in Figure 1

### 2.2 Advancing Spatiotemporal Approaches to Nutrient Availability Assessment

Understanding nutrient availability across diverse waste streams requires increasingly sophisticated analytical approaches that capture the dynamic, spatially heterogeneous nature of nutrient flows in complex agricultural and urban systems. Beyond aggregated assessments at administrative scales, emerging frameworks are revealing significant spatial and temporal variations that influence recovery feasibility and technology selection.

Nutrient recovery potential varies significantly not only by source type but also by specific waste stream characteristics within source categories.^80^ For example, poultry operations generate both solid litter and liquid manure with substantially different nutrient concentrations, moisture contents, and processing requirements. In another instance, confined animal feeding operations produce waste streams with varying compositions depending on animal type, housing systems, and management practices, requiring tailored recovery approaches for optimal efficiency.^81,82^

Given this complexity in waste stream characteristics and the need for tailored recovery approaches, recent advances in integrated manureshed frameworks (spatial systems for redistributing nutrients from concentrated sources to agricultural areas where they are needed) are expanding beyond the original county-level agricultural focus^83,84^ to incorporate municipal sources at watershed scales.^85^ This methodological evolution recognizes that nutrient recovery opportunities vary significantly across geographical and temporal scales. Municipal wastewater treatment plants represent substantial point sources that can fundamentally alter regional nutrient landscapes when considered alongside agricultural sources.^86^ Integration of agricultural and municipal sources at Hydrologic Unit Code (HUC) 8 watershed scale demonstrates that including municipal sources increases the total watersheds containing recoverable nitrogen sources by over 160% compared to agricultural sources alone and increases phosphorus source areas by 24%. It also reveals that over 70% of source areas are naturally adjacent to nutrient-deficient regions, creating spatial patterns that favor short-distance redistribution networks.^85^

The HUC8 watershed scale represents hydrologically coherent units averaging 1,800–2,200 km^2^ that align with natural nutrient transport pathways rather than administrative boundaries.^85,87^ This scale offers several advantages for integrated nutrient recovery analysis: watersheds represent natural topographic units where nutrient fate is governed by hydrologic processes^88,89^ the scale aligns with existing water quality management frameworks enabling integration with nutrient transport models, and many federal and state nutrient management programs already operate at watershed scales making results directly applicable to existing management structures.^85,90,91^ Additionally, integrating point-source municipal data with distributed agricultural sources is most appropriately conducted at watershed scales where cumulative impacts on receiving waters can be assessed.^87^

Supporting these expanded watershed-scale frameworks requires sophisticated data integration capabilities. Multimodal geospatial data integration enables comprehensive monitoring of crop growth, nutrient distribution, and hydrological dynamics across large-scale agricultural systems.^92,93^ However, integrating data at different scales requires advanced geospatial methods that consider resolution mismatches and different levels of precision in spatial datasets.^94^ The first step toward a robust and efficient data integration pipeline requires harmonizing and standardizing spatial data with Ontology-Driven FAIRification of data.^95,96^ Following this, datasets should be converted from latitude and longitude geocoding methods to Geohashes^97^ for precise, robust, and optimized data integration and merging processes. Geohashes are geocoding methods that represent geographic coordinates as short strings of digits and letters corresponding to specific areas on a map. This integrated result provides a detailed understanding of optimal deployment strategies for nutrient recovery technologies across different spatial and temporal contexts.

Geospatial phenomena are often dynamic, where environmental properties vary in time. Temporal dynamics present critical considerations for recovery system design and operations. Agricultural sources exhibit seasonal peaks related to animal housing cycles and manure application timing, while municipal sources maintain a relatively constant year-round availability, ensuring a stable supply for recovery operations. Spatial proximity analysis combined with temporal pattern recognition enables the optimization of recovery system design and operation strategies. In addition, spatiotemporal predictive models, such as spatiotemporal graph neural networks, can be used to forecast nutrient presence and concentration in land and water^98^ and provide additional insights for the implementation of recovery strategies. A comprehensive data integration framework can successfully monitor and quantify nutrient availability, considering fertilizer use, CAFO waste, agricultural runoff, and WWTP effects (Figure 2). This framework provides fundamental steps necessary for identifying recovery opportunities according to nutrient spatial distribution.

**Figure 2:**
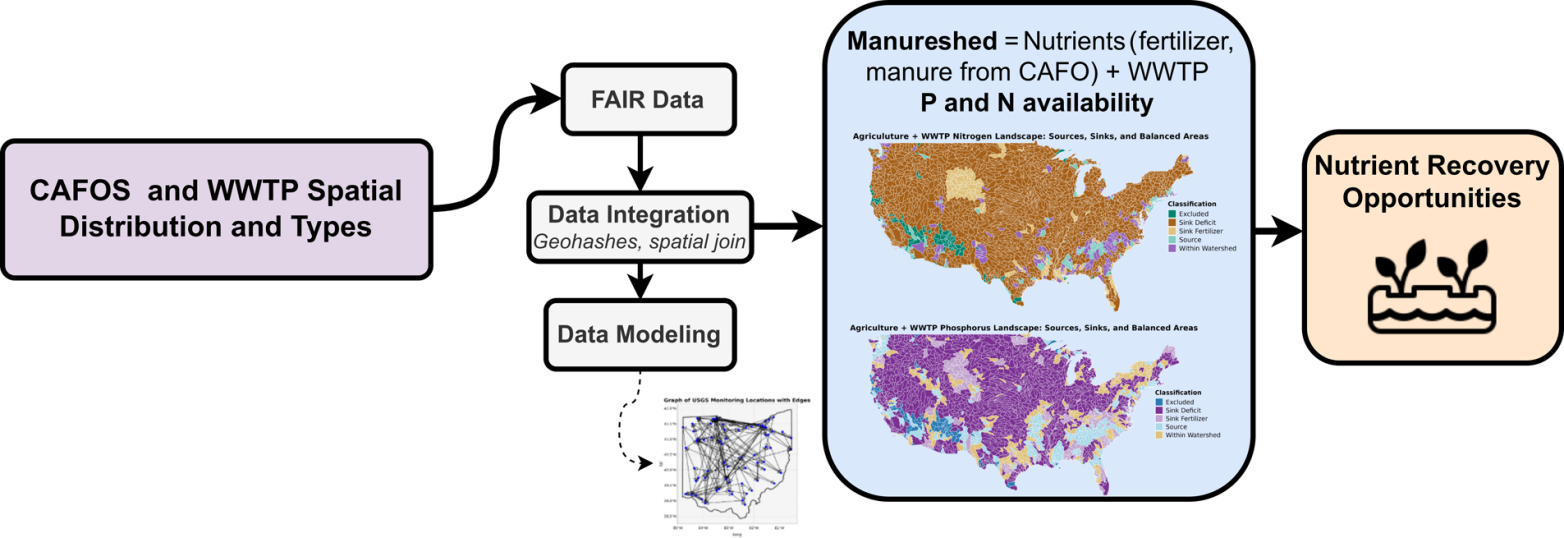
Spatiotemporal data integration framework for the nutrient availability assessment. Integration of fertilizer, WWTP, and CAFO data enables identification of nutrient recovery opportunities based on spatial distribution patterns.

Contemporary big data approaches reveal that optimal technology selection depends on multiple interconnected factors including waste stream composition, regional nutrient balances, seasonal variations, spatial proximity to potential users, and existing infrastructure capabilities.^92^ However, several significant obstacles remain for implementing robust spatiotemporal approaches to nutrient recovery. The sophisticated data integration and analytical capabilities required by these frameworks face substantial technical and logistical barriers. Data integration challenges include inconsistent temporal sampling frequencies across different monitoring systems, spatial resolution mismatches between satellite imagery and ground-based sensors,^93^ and limited availability of real-time nutrient concentration data from many agricultural and municipal sources.^99^ Additionally, computational scalability becomes problematic when processing high-resolution spatiotemporal data sets across continental scales, requiring substantial computational resources and optimized algorithms for practical implementation.^100^

Privacy and data sharing concerns present additional barriers, particularly when integrating proprietary farm management data with publicly available environmental datasets. Standardization issues across different data providers, varying quality control protocols, and the need for robust uncertainty quantification in predictive models further complicate comprehensive spatiotemporal analysis.^95^

Translating these spatiotemporal frameworks into operational nutrient recovery systems requires the address of specific technical gaps with quantifiable targets. First, standardized data exchange protocols must achieve temporal alignment within 24–48 h across agricultural, municipal, and environmental monitoring systems to enable responsive recovery operations that capitalize on seasonal nutrient availability peaks. Second, edge computing implementations should target a processing latency under 15 min for real-time optimization of recovery system operations, particularly critical for managing the 2.6× difference between total municipal nitrogen generation and effluent discharge that represents recoverable nutrients lost to biological treatment.^85,86^

Third, machine learning models designed for spatiotemporal nutrient prediction must achieve forecast accuracies exceeding 85% at weekly resolution and 75% at monthly resolution to support infrastructure investment decisions and technology deployment strategies.^98^ Given that transport costs represent the primary economic barrier to nutrient redistribution^101,102^ predictive models should prioritize subwatershed resolution (HUC10 or finer) to identify the short-distance transfer opportunities indicated by the >70% source-to-sink adjacency patterns observed at the HUC8 scale.^85^

Fourth, privacy-preserving data frameworks must balance granular spatial resolution (down to farm level for agricultural sources and facility level for municipal sources) with aggregated reporting that protects operational details while enabling regional nutrient flow optimization.^95^ Specifically, frameworks should enable nutrient availability forecasting at subwatershed scales while maintaining anonymity for individual operations through spatial aggregation and differential privacy techniques.

Finally, computational scalability targets should support continental-scale analysis processing daily updates across 2,000+ HUC8 watersheds within 6-h windows, enabling near-real-time identification of emerging recovery opportunities.^99,100^ This computational capacity is essential for managing dynamic nutrient flows where agricultural sources exhibit seasonal peaks while municipal sources provide year-round baseline availability.^85^ Integration of these quantitative targets into operational decision support systems will transform spatiotemporal nutrient assessment from research capability to a practical tool for maximizing recovery efficiency and economic viability across diverse waste streams and geographic contexts.

## 3 Current Nutrient Management Processes

Recent advances in wastewater treatment reflect a growing emphasis on both environmental protection and recovery of valuable nutrient resources. Current solutions fall into two broad categories. First, biological nutrient removal processes, already well-established in municipal WWTPs, remove nitrogen and phosphorus, but do not recover them. Second, physicochemical technologies, which are increasingly entering the commercialized realm, reclaim nitrogen and phosphorus nutrients in the form of fertilizers. The following subsections review both biological and nonbiological state-of-the-art technologies at full scale, focusing on their commercial deployment and practical considerations.

### 3.1 Biological Nutrient Management

To address excess nutrient discharge, municipal WWTPs employ biological secondary treatment processes to remove nitrogen and phosphorus contaminants.^7^ Biological nutrient removal (BNR) methods have distinct advantages over chemical and physical methods such as lower costs, higher efficiency, and an environmentally friendly effluent. Facilities with biological removal technologies achieve 85–95% removal of influent inorganic nitrogen while reducing concentrations to 3 mg-N/L and 0.5 mg-P/L.^8^

The most common nitrogen removal method is biological nitrification–denitrification (Figure 3).^103^ For nitrification, aerobic autotrophic bacteria oxidize ammonia to nitrite and then nitrate in a low-carbon environment, requiring 4.57 g-O_2_/g-N for total oxidation.^104^ Temperature, pH, and dissolved oxygen concentrations influence nitrification kinetics and are closely monitored. For denitrification, heterotrophic and autotrophic bacteria, mainly facultative anaerobes of the *Pseudomonas* species, reduce nitrate to nitric oxide (NO), nitrous oxide (N_2_O), and nitrogen gas (N_2_) at near-zero dissolved oxygen levels with a supply of carbon. Sources of carbon can be introduced from primary effluent recycle streams or external carbon sources such as methanol, ethanol, acetate, or glycerol.^103^ Denitrification kinetics are affected by temperature, pH, dissolved oxygen concentrations, and the specific carbon source.^105–107^ The most common process for nitrification–denitrification is the modified Ludzack–Ettinger (MLE) process, which implements a series of anoxic zones, followed by a final aerobic stage and clarifier. Often incorporating 3–4 stages in series for optimal efficiency, MLE can reach NO_3_–N concentrations of 4–7 mg/L given sufficient BOD and anoxic times (2–4 h).^104^

**Figure 3:**
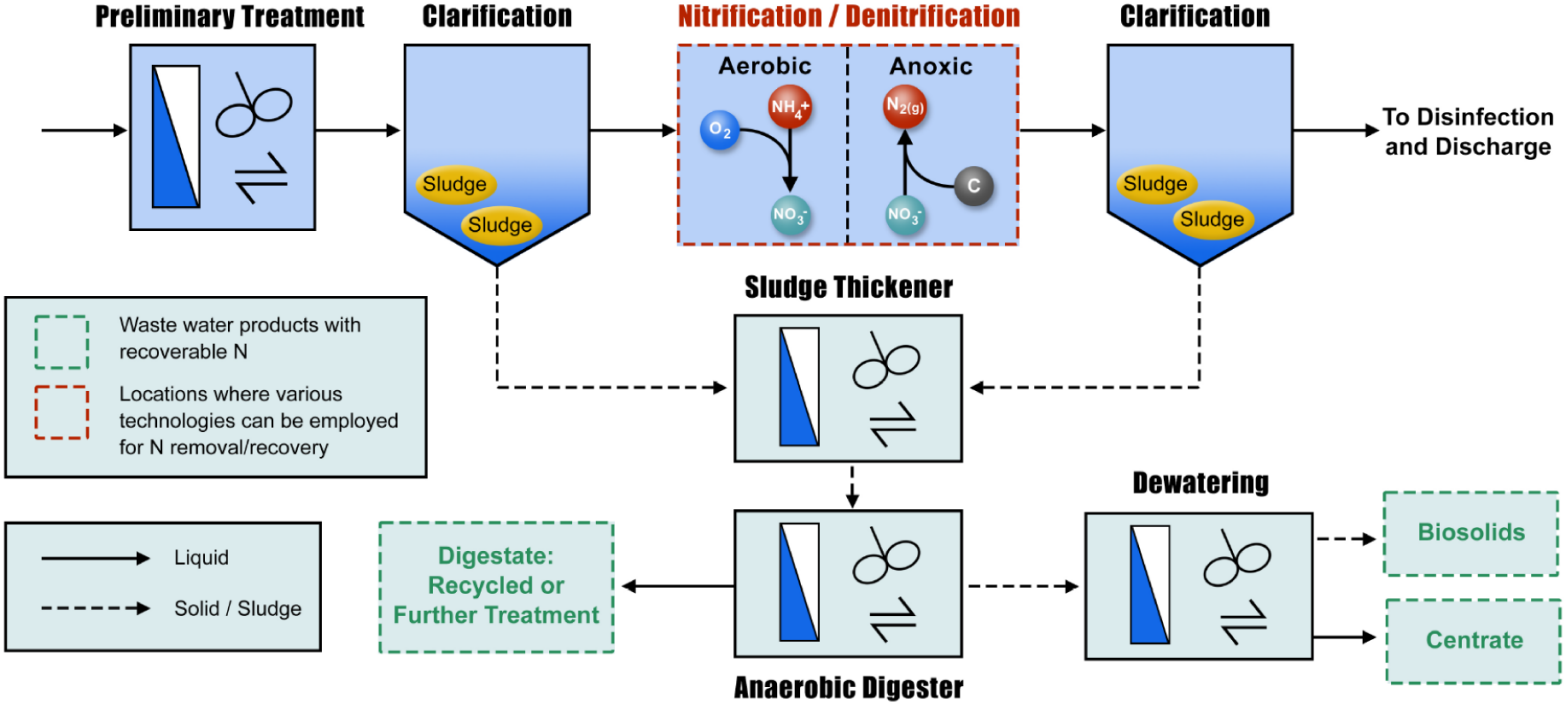
Simplified schematic of wastewater treatment with biological nitrification–denitrification.

Alternatives to the nitrification–denitrification process include nitritation–denitritation, deammonification, and physicochemical processes, which provide energy and carbon savings. Nitritation–denitritation removes nitrogen by partial nitrification of ammonia to nitrite (*nitritation*) followed by reduction of nitrite to nitrogen gas (*denitritation*).^108,109^ This partial nitrification leads to reduced oxygen demand, carbon requirement, and sludge produced. For example, the single-reactor system for High Ammonium Removal Over Nitrite (SHARON) reduces oxygen demand by 25% to 3.43 g-O_2_/g-N and carbon requirement by 40% to 2.4 g-COD/g-N using a 1-day retention time at temperatures above 30 °C.^110–112^ Deammonification directly converts ammonium to nitrogen gas using Anammox bacteria, which are anaerobic ammonium oxidizers belonging to the phylum *Planctomycetales*, in oxygen-limited conditions.^113^ In this process, ammonium acts as an electron acceptor when reacting with nitrite to produce nitrogen gas.^111^ Direct conversion of ammonium to nitrogen gas eliminates the need for dissolved oxygen and COD, and it reduces sludge production. Similarly to Anammox, iron-driven anaerobic ammonium oxidation (Feammox) and denitrifying anaerobic methane oxidation (DAMO) use iron-reducing bacteria and *Methylomirabilis*, respectively, for denitrification.^114,115^

Phosphorus removal methods are more recent. Due to key differences in the chemistry of nitrogen and phosphorus, simultaneous biological recovery has been an ongoing challenge and nitrogen is not typically sequestered. Phosphorus is typically present in wastewater as orthophosphate (PO_4_^3–^–P) or organic phosphorus.^9^

The primary biological nutrient sequestration technology employed at wastewater treatment facilities for phosphorus removal is enhanced biological phosphate removal (EBPR), which enriches PAO in activated sludge (Figure 4). PAOs accumulate phosphates under aerobic/anoxic conditions, but they also require anaerobic conditions to uptake and metabolize volatile fatty acids (VFA) prior to PO_4_^3–^ uptake.^116^ EBPR is therefore compatible with most full-scale activated sludge process configurations such as the common A^2^O process (MLE). Factors influencing EBPR performance include solid retention times (3–4 days),^8^ hydraulic residence times (typically 0.5–2 h for anaerobic and 4–12 h for aerobic),^117^ concentrations of nitrate/nitrite,^118^ carbon speciation, and specific VFA-to-phosphorus ratios (a ratio of 4–16 is recommended for VFA:total P).^8^ VFA concentrations are often insufficient for PAO growth and can be increased using side-stream fermentation or costly short-chain carbon supplements.^119,120^

**Figure 4:**
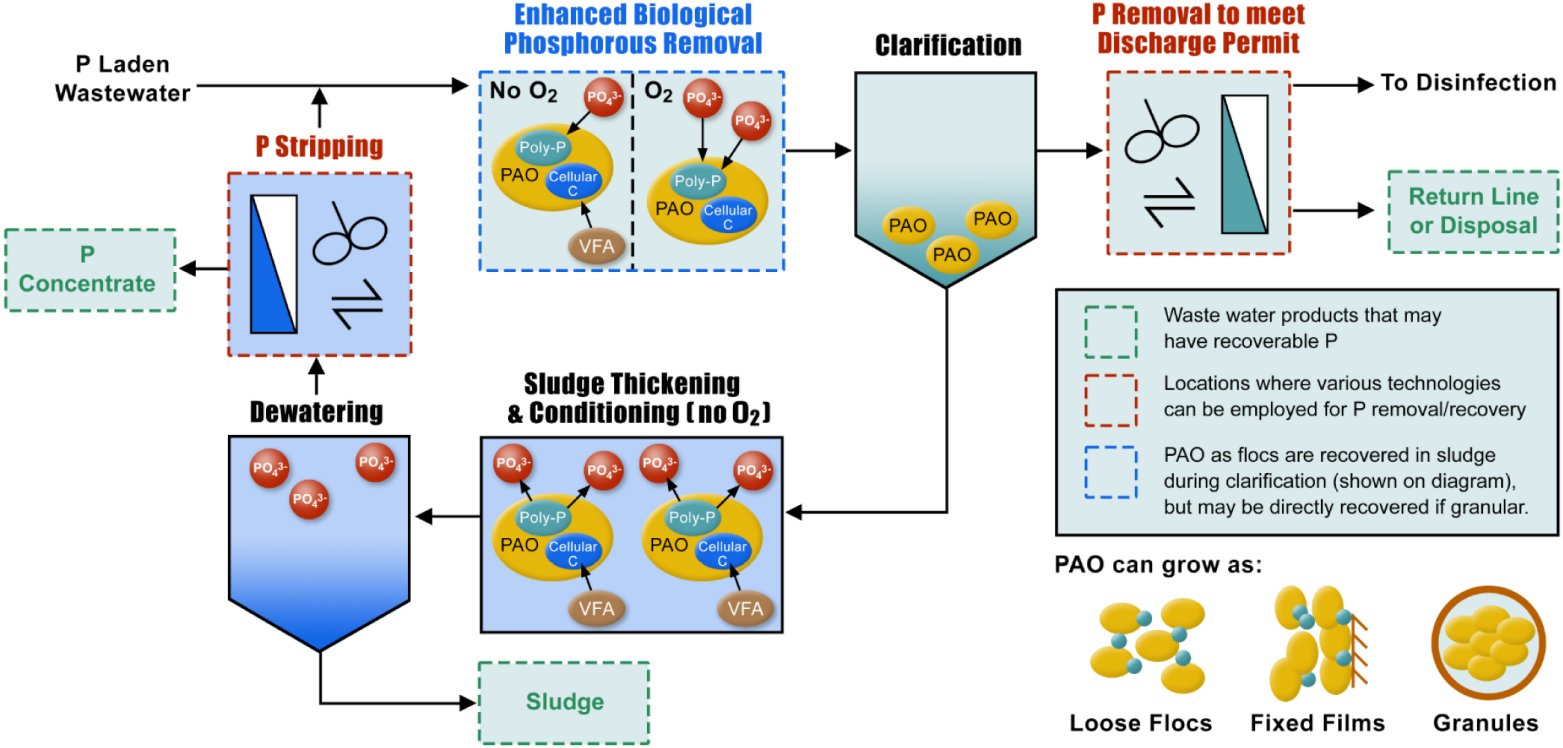
Simplified schematic of wastewater treatment with enhanced biological phosphorus removal (EBPR). Phosphorus is represented with blue in the flow diagram.

EBPR alone is often insufficient for wastewater treatment. For instance, EBPR can reduce PO_4_^3–^ to roughly 0.5 mg/L in wastewater effluent waters, but state discharge permit limits in the United States can be as low as 0.1–0.2 mg/L, PO_4_^3–^–P^8^ therefore requiring downstream phosphate removal using nonbiological processes. However, removal of phosphate through EBPR at concentrations less than 0.5 mg/L is less efficient compared to phosphate removal via membrane filtration and aluminum or iron flocculation.^121,122^ Phosphate accumulated in PAO is also subject to rerelease during the sludge wasting process under the low oxygen conditions typical of sludge digestion and dewatering.^9^ Phosphate must therefore be removed from sludge dewatering liquors prior to recirculation within the facility, which is not possible biologically without supplemental oxygen, additional VFA, and long retention times. Lastly, many PAOs are also denitrifiers, including recently identified incomplete denitrifiers that generate N_2_O from wastewater,^10^ suggesting that EBPR technology is unlikely to recover nitrogen but may contribute to nitrogen volatilization and greenhouse gas generation.

The phosphorus-laden sludge dewatering liquors produced during EBPR provide an additional opportunity to integrate physiochemical phosphorus removal processes into EBPR process trains (Figure 4). Existing and emerging technologies such as ion exchange resins and sorbents^123,124^ and (bio)­electrochemistry^125–129^ can capture phosphorus from these streams and are discussed in later sections. Struvite (MgNH_4_PO_4_·6H_2_O) precipitation is the rare example of wastewater technology that can sequester both nitrogen and phosphorus,^130^ and struvite (bio)­precipitation can be employed on sludge dewatering liquors.^131^ Struvite formation is most promising in concentrated phosphorus streams, as struvite removes nitrogen and phosphorus in a 1:1 molar ratio, while the N:P ratio in dewatering liquors is much higher.^132^

A range of nutrient concentrations, BOD, and pH values can be estimated based on flow diagrams of common WWTPs (Table 2). Together with Figures 3 and 4, we can see that the concentrate and sludge from the dewatering process have high concentrations of nitrogen (204–1,010 mg-N/L) and phosphorus (85–255 mg-P/L), demonstrating opportunities for nutrient recovery without altering existing unit operations. Secondary treatment process stages using biological nutrient management methods would be the key location where nutrient recovery technologies could be employed, as this is where a maximum of 90% nitrogen removal and 95% phosphorus removal takes place. For more rigorous nutrient management, an additional unit was introduced prior to discharge. However, this would require advanced separation technologies efficient at dilute concentrations of 3–16 mg NH_4_–N/L and 0.5 mg-P/L. Thus, an effective strategy for enhancing nutrient recovery in municipal WWTP would initially target the sludge and centrate streams produced during the dewatering process, as these require minimal modifications to existing infrastructure. Subsequently, dedicated recovery technologies should progressively replace conventional biological nutrient removal processes, such as nitrification–denitrification and EBPR, to maximize the recovery of nitrogen and phosphorus from wastewater.^133^ Finally, advanced separation technologies optimal at dilute concentrations would focus on recovering the remaining nutrients to meet the discharge limits of WWTP effluents.

**Table 2:** Municipal WWTP Liquid Stream Characteristics Calculated Based on Removal Efficiencies of Unit Operations^a^

WWTP Stream (−)	TKN^b^ (mg/L)	NH_4_^+^–N (mg/L)	Total P (mg/L)	BOD (mg/L)	**pH** (−)
Influent	20–85	12–60	5–15	100–360	6.5–8.0
Preliminary Treatment Effluent	20–85	12–60	5–15	100–360	6.5–8.0
Primary Clarifier Effluent	16–68	9–48	4–9	60–250	6.5–8.0
Secondary Treatment Effluent	5.5–23	3.3–16	<0.5	12–50	7.0–7.5
Primary Sludge	368–1,562	221–1,094	115–345	3,310–11,900	5.5–8.0
Secondary Sludge^c^	60–252	36–176	5–7	136–567	6.6–8.0
Digestate	40–166	24–116	13–39	n/a	6.5–8.0
Centrate	340–1,445	204–1,010	85–255	n/a	6.5–8.0
Discharge	<3.0	1.5–2.5	<0.5	12–50	7.0–7.5

a Sludge streams with a significant amount of supernatant were also included.^103,134–136^

b TKN: total Kjeldahl nitrogen.

c Concentrations can differ based on recycle streams and nature of secondary treatment.

### 3.2 Physicochemical Nutrient Management

Various physicochemical nutrient management processes provide product streams of value through nutrient recovery, leading to notable advancements in their commercialization. Most commercial installations focus on WWTPs, landfills, and CAFOs, primarily targeting the recovery of nutrients in their crystalline form. In addition, the integration of multiple physicochemical separation technologies opens opportunities to target multiple commercial products such as clean water, concentrated fertilizer products, biosolids, and biogas (e.g., methane).

The main, nonbiological-based technologies at scale for nutrient recovery are air stripping (nitrogen recovery) and crystallization (phosphorus recovery) (Table 3).^137–148^ Among the three, air stripping has been the most widely commercialized technology. Byoflex implements air stripping to handle feedstock that is up to 15% dry matter and requires no prior filtration.^141^ Task Environmental Engineering’s urban and industrial wastewater treatment process utilizes an air stripping tower combined with an ammonia absorber, allowing the production of nitrogen-based salt fertilizers.^146^ Additionally, Task Environmental Engineering recycles the incoming gases and foregoes the use of steam to lower energy costs. Another company, Mach Engineering, markets their stripping technology for purifying wastewater streams of organic contaminants.^145^ With low pressure drops within the air strippers, they are durable and serve as a part of a larger waste treatment system.

**Table 3:** Current Physicochemical Nutrient Recovery Commercial Installations

Company Name	Technology	Company Footprint	Feedstock(s)	Product(s)	Nutrient Recovered
Organics Group^140^	Air stripping	Est. 1992, unknown number of installations	Landfill leachate	Ammonia-rich streams	N
Byoflex^141^	Air stripping	Est. 2011, 32 listed projects	WWTP, manure, food waste	Ammonia-rich streams	N
Hansa Eng.^155^	Air stripping	Est. 1954, RVT Process Equipment tech.	Ammonia-contaminated stream	Ammonia-rich streams	N
Heil Eng. Process Equipment^143^	Air stripping	Est. 1929, general air-stripper vendor	Ammonia-contaminated stream	Ammonia-rich streams	N
Indusco Env.^144^	Air stripping	Est. 1970s, general ammonia-stripper vendor	Ammonia-contaminated stream	Ammonia-rich streams	N
Mach Eng.^145^	Air stripping	Est. 2005, unknown number of installations (equipment rental)	WWTP, energy applications, utility applications	Ammonia-rich streams	N
GNS^146^	Air stripping	Est. 1998, 2-unit installation with Systemic	Corn silage and manure digestate	Biogas and ammonium sulfate fertilizer	N
Nijhuis Industries^147^	Air stripping	Est. 1904, general ammonia-stripper vendor	Digestate	Ammonia-rich streams	N
RVT Process Equipment^142^	Air stripping	Est. 1976, main equipment manufacturer	WWTP streams	Ammonia-rich streams	N
Task Env. Eng.^146^	Air stripping	Est. 1988, main equipment manufacturer	WWTP streams	Ammonia-rich streams	N
Bion Env. Tech.^156^	Thermal and mechanical	Est. 1987	CAFO cattle manure waste streams	Clean water, natural gas, fertilizer	N
CNP Cycles (AirPrex/LysoPhos)^156^	Crystallizer, thermal hydrolysis	Est. 2011, 10 installations (AirPrex and LysoPhos)	WWTP streams	Struvite	N, P
NuReSys^138^	Crystallizer, pH air stripping	Est. 2011, 7 commercial systems installed	Industrial/municipal waste streams (centrate, digestate, hybrid)	Struvite	N, P
Ostara^139^	Crystallizer	Est. 2005, 23 commercial installations worldwide	WWTP streams	Struvite	N, P
N2 Applied^151^	Electrolyzer	Est. 2010, 10 projects installed	Animal manure, air, electricity	Nitrogen fertilizer	N
ReMo Energy Inc.^157^	Electrolyzer	Est. 2020, MOU signed with Trammo	Air, water, renewable energy	Nitrogen fertilizer	N
Sedron Tech.^150^	Thermal	Est. 2017, handles 15.6 million gal manure/yr	WW biosolids, sidestreams, dairy waste, LCFS digestate, raw septage	Clean water, dry fertilizer, aqueous ammonia	N
Digested Organics^8^	Membrane filtration	Est. 2013, 20+ commercial installations	Manure, digestates, food/beverage, industrial/municipal, landfill	Biogas, biofertilizer	N, P

The most notable company that implements struvite crystallization is Ostara with over 23 commercial installations globally.^139^ Recovery of nutrients via crystallization is beneficial, especially for WWTP systems, as it reduces the extent of nutrient crystallization in pipelines, allowing for decreased maintenance at locations within the WWTP. The struvite produced by Ostara is termed the Crystal Green Pearl fertilizer, which contains a nitrogen–phosphorus-potassium (NPK) mass ratio of (5–28–0) and 10% magnesium. It should be noted that Crystal Green Pearl’s NPK mass ratio differs from typical struvite NPK (6–29–0) due to potential impurities mixed in the fertilizer blend.^149^

Thermal, electrochemical, and membrane filtration technologies have also been commercialized at large scale. Sedron Technologies uses thermal recovery technology and makes use of a wide range of feedstocks such as wastewater biosolids, wastewater sidestreams, dairy waste, low-carbon fuel standard digestate, and raw septage.^150^ The products are clean water, dry fertilizer, and aqueous ammonium of approximately 10 N wt %. Digester Organics is a rare commercial company that uses membrane filtration.^8^ The company takes wastewater from dairy and swine farms, distilleries, food/beverage, breweries, industrial digesters, and landfill/composters and converts them to products, such as biogas and biofertilizer. N_2_ applied makes fertilizers from liquid organic substrates by using plasma technology to convert electricity and air into reactive nitrogen gas, which absorbs to the organic matter.^151^ This enriches the nitrogen content and prevents a severe loss of ammonia from the fertilizer. As shown, many commercial installations are recovering and valorizing nutrients from wastewater. However, high energy consumption, production of greenhouse gases, addition of chemicals, reliable product quality, and large process footprints discouraging modular system installations are significant disadvantages of commercialized physicochemical processes.^152–154^ The following sections expand on the mechanism and technological progress of these technologies and future directions to mitigate their disadvantages.

## 4 Conventional Nutrient Recovery Technologies

Air stripping, struvite precipitation, and ion exchange have been tested for nutrient recovery purposes for decades at large scales.^158^ Advancements in materials, improved understanding of fundamental mechanisms, and testing with diverse wastewater feedstocks have directly increased achievable concentration factors, percent recovery, and the nutrient content of the final product using conventional recovery technologies. As a result, contemporary commercialization installments often focus on such technologies. The following sections review the fundamental mechanisms, performances, and future directions for crystallization, ammonia stripping, and adsorbent/ion exchange of nitrogen and phosphorus nutrients.

### 4.1 Crystallization and Precipitation

Struvite precipitation is one of the main commercial methods used to recover phosphorus and nitrogen from wastewater. Struvite (MgNH_4_PO_4_·6H_2_O) is a white crystalline mineral applied as a slow-release fertilizer, which reduces the extent of nutrient runoff.^159,160^ The direct production of struvite from wastewater allows the simultaneous recovery of phosphorus and ammonium (eq 1) and presents an economically attractive product for conventional wastewater treatment processes.^161–163^

1 $$Mg^{2+}+N{H_{4}}^{+}+P{O_{4}}^{3-}+6H_{2}O\to MgNH_{4}PO_{4}\cdot 6H_{2}O$$

Struvite crystallization is promoted by increasing the saturation index (SI) above zero (eq 2). SI governs the productivity of struvite precipitation by influencing the regulation of nucleation, crystal growth, and agglomeration rates, which heavily affect the amount of alkaline and magnesium chemicals to be added.

2 $$\mathrm{SI}=\log\left(\frac{\prod a_{i}}{K_{s(\mathrm{str})}}\right)=\log\left(\frac{a_{{\mathrm{Mg}}^{2+}}a_{{\mathrm{NH}}_{4}^{+}}a_{{{\mathrm{HPO}}_{4}}^{2-}}a_{{\mathrm{OH}}^{-}}}{K_{s(\mathrm{str})}}\right)$$

For struvite, the SI value relies mainly on the solubility product of struvite *K*_s(str)_ (ranges from 1.15 × 10^–10^ to 7.59 × 10^–14^ at 25°C) and the ionic activities *a*_*i*_, which resemble ion concentrations.^161,164^ Excessively high concentrations of magnesium and phosphorus lead to oversaturation, which reduces the crystal size and increases the settling time and percent recovery.^161,165^ Various factors influence the yield of struvite, including pH, saturation index, temperature, reactor type, and the concentration of dissolved elements such as calcium (Ca^2+^) and carbonates (CO_3_^2–^) that can inhibit struvite formation.^161,162^ As a result, preliminary treatment steps such as anaerobic digestion, electrocoagulation, chelation, microwave treatment,^166^ and culture with peptides^167^ are necessary to maximize phosphorus recovery.

The main barriers for struvite precipitation are operational costs (energy costs for mixing and pumping), chemical additives for adjustment of pH and magnesium molar ratio, and recovery efficiency at low nutrient concentrations.^168,169^ Operational and chemical costs together represent up to 75% of the total treatment cost, acting as the main economic barrier.^170,171^ Promising solutions that address these cost issues include implementing renewable energy^78^ and careful selection of the magnesium source and dosing. Recovery efficiencies can drastically decrease when feedstock concentrations are low or a sufficient concentration is not achieved prior to precipitation. Low concentrations translate to being limited by equilibrium solubility and requiring more resources to saturate the solution. More minor limitations include fouling and scaling on surfaces of the reactor and maintaining a stable operation despite varying wastewater characteristics.

To address struvite precipitation viability with wastewater, studies have used various substrates, including sewage sludge and different types of manure, with poultry manure showing the highest content of recyclable phosphorus (82%).^172^ Commercial technologies have successfully achieved approximately 80% phosphorus removal, generating struvite at rates ranging from 0.89 to 13.7 kg per kilogram of influent phosphorus. MgCl_2_, MgO, MgSO_4_, Mg­(OH)_2_, and seawater^173^ have served as magnesium sources (Table 4). The type of crystallizer used, which is mainly either stirred reactors (SR) or fluidized bed reactors (FBRs) with air or liquid as fluidizing agents, also influences the effectiveness of struvite precipitation. SRs are simple to operate and often achieve high P-extraction yields, while FBRs produce larger particles but are more expensive to operate as a result of recirculation flow. Both configurations produce fines (small, unwanted crystals) due to the high mixing and fluidization speed.^161^ Electrochemical methods of struvite crystallization using sacrificial magnesium electrodes have also been explored,^174–176^ showing opportunities of using novel reactors for crystallization.

**Table 4:** Performance Summary of Commercial-Scale Technologies for Struvite Production^173^ ^a^

Technology	Reactor Type	Removal Efficiency (%)	Normalized Production Rate (kg final product/kg influent P)	Total Cost ($/kg P recovered)	Mg Source
Ostara	FBR	85	0.89–10	10.3	MgCl_2_
Multiform	FBR	80–90	2.3–5	5.0	MgCl_2_
PHOSNIX	FBR	90	7–8.5	3.24	Mg(OH)_2_
AirPrex	Stirred	80–90	2–10	8.14	MgCl_2_
PHOSPAQ	Stirred	80	2.3–8.3		MgO
NuReSys	Stirred	85	3.3–13.7		MgCl_2_
ANPHOS	Stirred	80–90	7.1–7.8		MgO/Mg(OH)_2_

a Reproduced from Ghosh, S., Lobanov, S., and Lo, V. K., An overview of technologies to recover phosphorus as struvite from wastewater: advantages and shortcomings. Environmental Science and Pollution Research, 2019, 26(19), 19063–19077, with permission from SNCSC.

Other forms of crystallization products from wastewater are also possible. For example, K-struvite (MgKPO_4_·6H_2_O) is a valuable fertilizer source recovered similarly to struvite with potassium (K), another essential nutrient in many fertilizers, replacing NH_4_^+^.^177,178^ Alternatively, various forms of calcium phosphate can be obtained by chemical precipitation, taking advantage of the calcium present in most waste streams. Calcium phosphate (CaP) compounds such as hydroxyapatite (HAP, Ca_5_(PO_4_)_3_OH), dicalcium phosphate dihydrate (DCPD, CaHPO_4_·2H_2_O), dicalcium phosphate anhydrate (DCPA, CaHPO_4_), tricalcium phosphate (TCP Ca_3_(PO_4_)_2_), and amorphous calcium phosphate (ACP Ca_3_(PO_4_)_2_) can be used as P fertilizers, but their availability for plant uptake varies according to solubility. Of these calcium phosphate compounds, DCPD exhibits the highest solubility, followed by TCP, ACP, and HAP, with TCP and ACP showing comparable solubility levels.^179^

To maximize the crystal yield and product purity, a more thorough understanding of competing precipitation chemistry in wastewater conditions is required. Also, further optimization of control over crystal morphology will be necessary because the size and shape of slow-release fertilizers are known to have an impact on the rate of nutrient release.^180^ Integration with other unit operations also holds promise for crystallization. Studies on the integration of precipitation and adsorption techniques demonstrated an improved percentage of recovery, utilizing cost-effective adsorbent materials (particle size 50 μm) like biochar, diatomite, and zeolite as seeding materials. The size of the struvite crystal reached at least 100 μm, which is favorable for optimal struvite collection and nutrient uptake by plants.^181^

### 4.2 Ammonia Stripping for Nutrient Separations

Ammonia stripping is a widely used ammonia recovery technology that exploits the thermodynamic equilibrium (*K*_a_) between ammonium (NH_4_^+^) and ammonia gas (NH_3_) (eq 3).^182–187^

3 $$K_{a}=\frac{\left[NH_{3}\right]}{\left[N{H_{4}}^{+}\right]\left[OH^{-}\right]}$$

The ammonia stripping process involves four main steps: 1) conversion of ammonium ions to ammonia, 2) diffusion of aqueous ammonia to the air–water interface, 3) release of ammonia into the air interface through volatilization, and 4) diffusion of ammonia from the air–water interface to air.^188^ Conversion is promoted by adding alkali chemicals prior to phase change to increase the pH, such as NaOH, Ca­(OH)_2_, or CaO. After ammonia diffuses into the air phase, it is absorbed by an acid solution, most commonly H_2_SO_4_, and collected in the form of ammonium sulfate ((NH_4_)_2_SO_4_).

This technique stands out for its simplicity and cost-effectiveness, and it has been extensively tested in pilot and full-scale applications in WWTP and CAFOs waste.^141–147,155^ In terms of operation energy consumption, the energy consumption for ammonia stripping (6–13 kWh/kg-N) is comparable to the Haber–Bosch (10–13 kWh/kg-N) process.^182,189^ Although dependent on the retention time and operating parameters, removal percentage up to 95% can be reached in 12 h.^187–192^

However, ammonia stripping for large-scale applications has several limitations such as CO_2_ emissions trapped in carbonic acid derivatives and emitted into the atmosphere,^193^ waste heat,^194^ and cost of chemicals.^195,196^ Of those, the cost of chemicals represents a significant constraint. The p*K*_a_ of ammonia is 9.2–9.3, requiring vast amounts of alkaline chemicals to maintain a pH greater than 10.5 or 12. Large volumes of sulfuric acid are also needed to store the stripped ammonia. This necessitates careful pH control throughout operation to minimize the amount of chemical addition.^197^ Additionally, significant waste heat is lost, which lowers the efficiency of this thermal technology. Integrating rigorous waste heat recycling will greatly improve the economic feasibility.^193,198^

To address these issues and maximize the recovery rate, numerous reactor architectures for stripping have been explored (Figure 5). Starting from the basic design that takes advantage of simple thermal volatilization, packed-bed columns and bubble aeration columns have been incorporated to increase the superficial area in contact between the stripping gas and the solution. These approaches force the stripping gas to flow in contact with the liquid or a wet-wall column. With such design optimization, ammonia stripping can achieve up to 98% removal.

**Figure 5:**
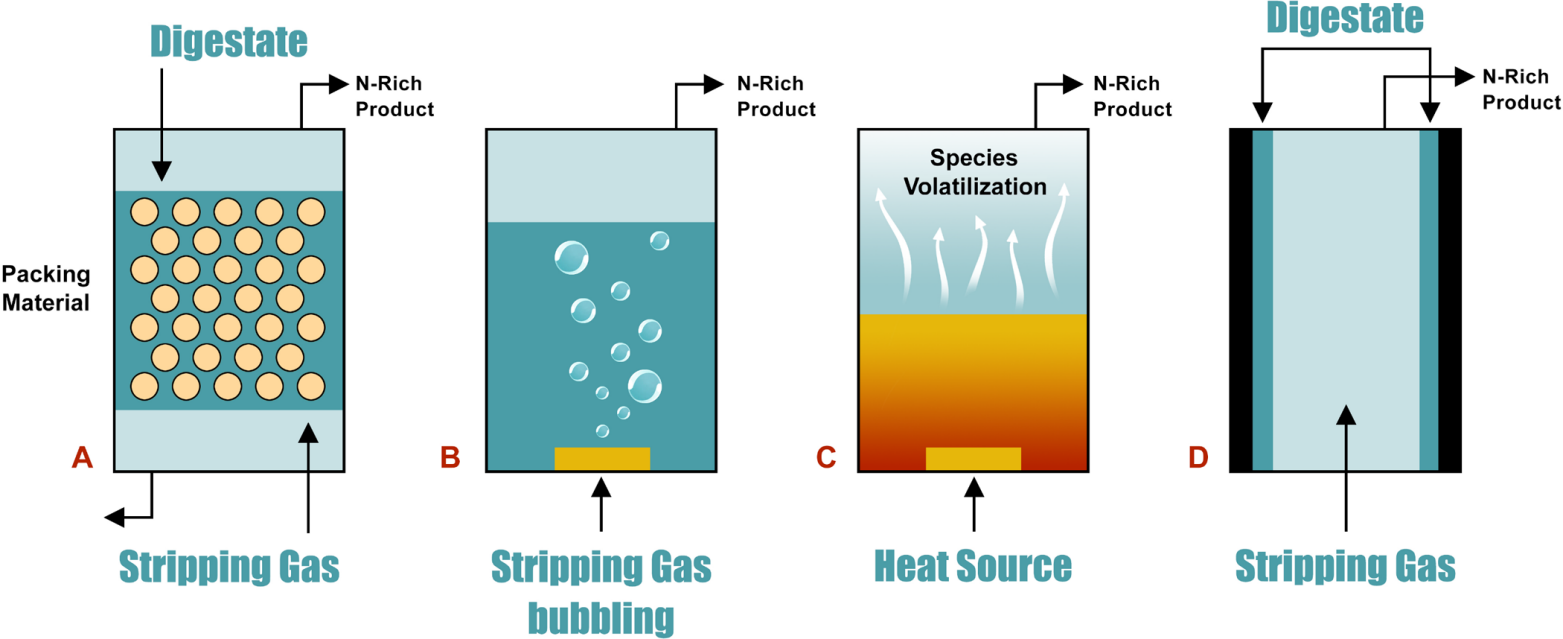
Schematic representation of different stripping column designs: a) packed bed column, b) bubble aeration column, c) thermal stripping, and d) wet-wall column.^187^ Reprinted from Renewable and Sustainable Energy Reviews, 143, Palakodeti, A., Azman, S., Rossi, B., Dewil, R. and Appels, L. A critical review of ammonia recovery from anaerobic digestate of organic wastes via stripping, 110903, Copyright 2021, with permission from Elsevier.

Increasing the temperature of the ammonia-containing solution can assist the process, but maintaining a pH level above 10.5 is critical to effectively remove ammonia. The introduction of vacuum in various thermal stripping configurations allowed removal and recovery rates to exceed 90%, where vacuum pressures ranged from 5.9 to 8.8 kPa and temperature ranged from 78 °C to 92 °C.^199^ Another approach improved efficiency by introducing wastewater feed as droplets, maximizing surface area for mass transfer.^200^ This technique yielded an ammonia removal rate of approximately 85% while maintaining recirculating feed temperatures at 55 °C. The ammonia recovered is often repurposed as ammonium sulfate at concentrations around 35 wt % (7.35 N wt %) to prevent crystallization, which becomes problematic at levels higher than 40 wt %.

### 4.3 Adsorbents and Ion Exchange Processes for Nutrient Separations

Adsorbents for ammonium nitrogen recovery from liquid waste are often classified by the type of material. Common types of adsorbents include minerals (e.g., zeolites, bentonites, and clays), activated carbon, polymers, industrial byproducts (e.g., biochar), and synthesized nanoparticles. Among these types, natural mineral zeolites are widely used for nitrogen recovery (Figure 6A)^19,201–206^ due to their selectivity for sorption, sorption capacity, environmental sustainability, structural stability, scalability, widespread availability, affordability, and regeneration capacity.^207,208^ Ammonia nitrogen adsorption by zeolites (ZO) occurs predominantly through the selective ion exchange (eq 4) mechanism.

4 $$ZO\cdot Na^{+}+N{H_{4}}^{+}\to ZO\cdot N{H_{4}}^{+}+Na^{+}$$

**Figure 6:**
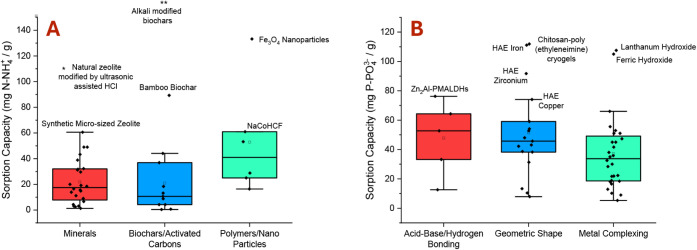
Sorption capacities of A) ammonia nitrogen reported in the literature using minerals, biochars/activated carbons, and polymers/nanoparticles, and B) phosphate phosphorus reported in the literature using acid–base/hydrogen bonding, geometric shape, and metal complexing approaches at pH range 4–6. Outliers are indicated by * and **.^19,201,203,204,211–220^ * 111.11 mg N–NH_4_^+^/g natural zeolite modified by ultrasonic-assisted HCl.^220^ ** 300–500 mg N–NH_4_^+^/g alkali-modified biochar.^221^

The mechanism involves the replacement of structural cations with dissolved ions in the surrounding environment. Typical adsorption capacities for zeolites range from 3 to 30 mg of NH_4_–N/g zeolite,^209^ with natural zeolite modified by ultrasonic-assisted HCl treatment reaching up to 111.11 mg NH_4_–N/g zeolite (10 N wt %).^203^ In general, synthetic zeolites and sodium-activated naturally occurring zeolites outperform nonpretreated natural occurring materials.^210^

Important performance metrics of adsorbents for nutrient recovery include the long-term sorption capacity, removal percentage, and regeneration efficiency. Long-term sorption capacity is defined as whether the material can endure at least 10 adsorption/regeneration sequences. Zeolites tested at pilot scale reported up to 62 cycles in a WWTP using centrate from anaerobic digestion sludge as feed.^222^ High removal is defined as when NH_4_–N concentration downstream of the adsorption process is <1 mg of NH_4_–N/L with removal percentage between 40 and 100%, depending on the inlet concentration.^19^ Efficient regeneration is accomplished if the adsorption capacity of the material can be regenerated or restored to its original state after the loading step; it is typically expressed as the percentage of the original adsorption capacity that is restored during the regeneration process, which is typically in the range of 70–100% for zeolites.^223,224^

While the utilization of zeolites is a well-established approach for removing and concentrating nitrogen in the regenerant brine stream, challenges exist when reusing the nitrogen-concentrated brine as fertilizer due to its low nitrogen concentration, which is typically between 1 and 5 g NH_4_–N/L (0.1–0.5 N wt %).^19,224^ This falls below the minimum levels of typical nitrogen-based fertilizer, which contain a minimum of 1.5 and 10 N wt %.^225^ To tackle this limitation, ion exchange was integrated with other processes. For example, hollow fiber membrane contactor (HFMC) stages enable separation and concentration of NH_4_^+^ from the zeolite stage brine to produce a 10–15% NH_4_^+^ stream after the HFMC closed loop.^201^ Air stripping processes have also been successfully integrated with ion exchange adsorption loops at pilot scale to produce ammonium sulfate fertilizer with high nitrogen recovery percentage of roughly 80% from the centrate of anaerobic digestion sludge.^222^

Beyond zeolites, various other adsorbents have been studied (Figure 6A), including other minerals like bentonite and clays, biochars and activated carbons, and synthesized polymers and nanoparticles.^19,213,217,226^ , However, these adsorbents do not have a clear material regeneration and nitrogen recovery methodology and have thus been mostly applied as single-use nitrogen removal processes.

Adsorption has also become a promising method for phosphorus removal from wastewater due to low costs, minimal waste products, and ease of operation without additional sludge production.^227^ As such, various adsorbents for the removal of phosphorus have been developed over the past decade to increase selective sorption rate,^228^ including anion exchange resins,^229–232^ iron oxide-based adsorbents,^233^ aluminum-containing materials,^234^ and double-layered hydroxides.^235^ However, the selective sorption of phosphorus is mainly challenged by the high hydration energy of phosphate. To circumvent this limitation, approaches in altering the acid–base properties, geometric shape, and metal complexing ability (Figure 6B) have been explored.^228^ Acid–base approaches for phosphorus selectivity tend to utilize H-bond acceptor groups to attract protonated phosphate anions. Approaches focused on geometric shapes result in the development of synthetic molecularly imprinted polymers with binding sites specific to phosphorus-containing molecules.^228^ Lastly, approaches focused on metal complexing ability have resulted in the formation of metal-based sorbents, in which ligands on the surface are replaced by another ligand, such as phosphate, in an aqueous exchange process.^236,237^ For example, a magnesium oxide-doped ordered mesoporous carbon sorbent enhances the electrostatic attraction, ligand exchange, and Lewis acid–base interactions for phosphate adsorption.^236^ In addition, exposing specific metal oxide crystal faces alters the number and arrangement of active sites, which serves as a strategy for achieving high selectivity for phosphorus.^238^ Higher adsorption capacities of phosphorus directly correlate to enhanced phosphorus nutrient recovery through struvite precipitation.^239,240^

### 4.4 Membrane Distillation

Membrane distillation (MD) is a thermally driven membrane separation technology that relies on the vapor pressure gradient or the concentration gradient of ammonia as the primary driving force for mass transfer.^241^ Volatile components such as ammonia are transported across a hydrophobic membrane contactor, where it is recovered.^242–244^ Membranes for membrane distillation are often constructed from hydrophobic materials such as polytetrafluoroethylene (PTFE), polypropylene (PP), and poly­(vinylidene fluoride) (PVDF), facilitating the passage of distillate while containing the retentate.

The hydrophobic membrane inhibits the physical mixing of the two media, while allowing the mass transport of volatile compounds to cross the pores of the membrane. There are four primary approaches for MD technologies: direct contact (DCMD), air gap (AGMD), sweeping gas (SGMD), and vacuum (VMD), each with different mass and energy transfer benefits (Figure 7).^245^ DCMD has the benefit of direct contact with a cold stream that can have partial selectivity for only ammonia, since the partial vapor pressure of the water is the same on both sides. AGMD and SGMD give moderate selectivity and relatively slow mass transfer.^246^ AGMD is used mainly for desalination as the air gap allows for a condensate film of vaporized water.^247^ As for the VMD, it shows the highest amount of mass transfer but has the lowest selectivity.^246^

**Figure 7:**
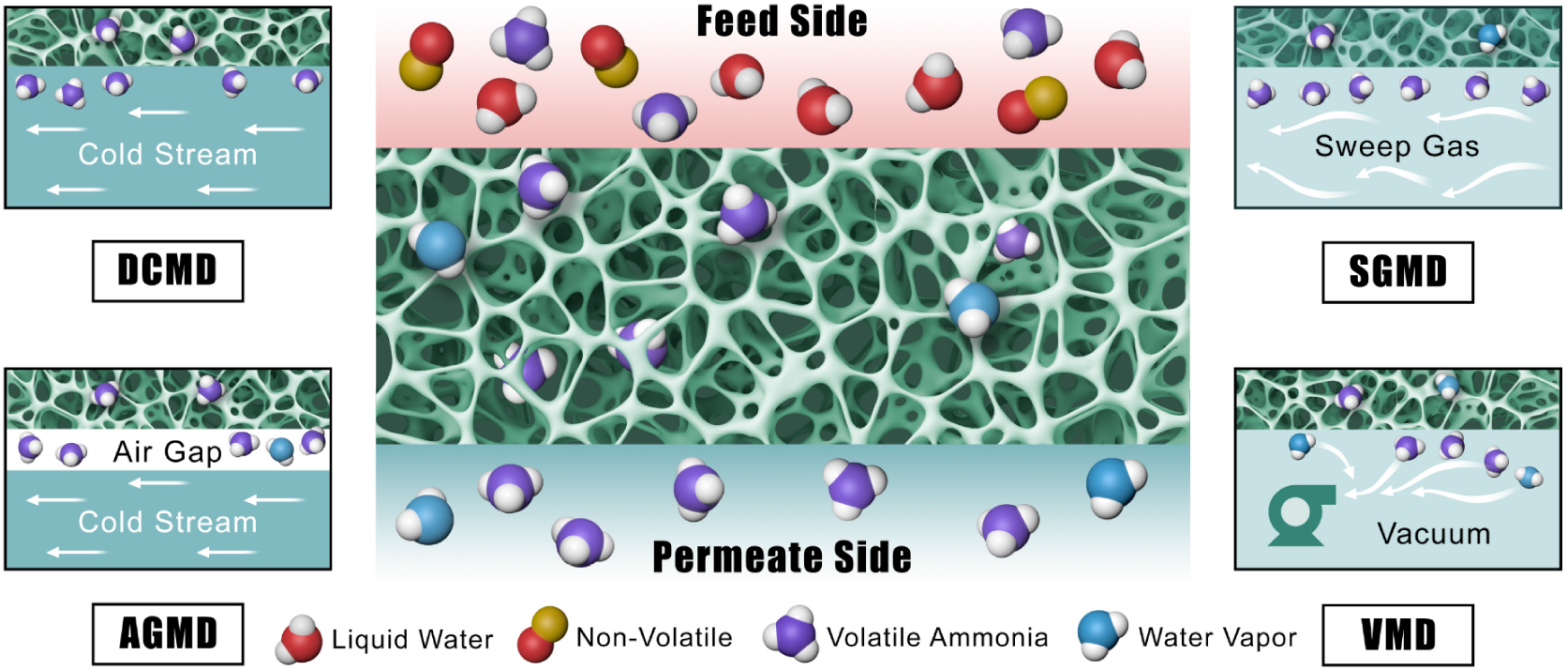
Schematic of different MD setups: DCMD, AGMD, SGMD, and VMD.^248^ Reprinted from Journal of Membrane Science, 616, Anvari, A., Yancheshme, A. A., Kekre, K. M., and Ronen, A., State-of-the-art methods for overcoming temperature polarization in membrane distillation process: A review, 118413, Copyright 2020, with permission from Elsevier.

Regardless of the configuration used, the efficiency of the separation process is influenced by a number of factors such as membrane properties (porosity, thickness, and hydrophobicity), temperature, and concentration polarization.^242,244,249^ Membrane properties influence the mass transfer of the process as it can lead to pore wetting, temperature polarization, and concentration gradient, ultimately reflecting the flux of volatile components. Pore wetting can sometimes also be caused by condensation of water inside the membrane, lowering the mass transfer force by 40–65%.^248,250^

The pressure across the membrane, strongly influenced by the pore size and thickness, is the main factor affecting mass transfer followed by temperature and flow velocity.^242^ Introduction of a localized heating membrane enhances the mass transfer while decreasing thermal energy consumption.^250^ The membrane is heated through Joule heating (e.g., electrical heating) next to the membrane’s surface. These approaches use heat sources of carbon-based materials such as reduced graphene oxide or carbon nanostructures coated on hydrophobic surfaces.^245,251,252^ The approach of Joule heating at the membrane surface also helps prevent membrane wetting by clearing pathways for vaporized molecules driven by the thermal profile of the membrane.^253^ This understanding is crucial when considering the optimization of the technology’s energy consumption, notably emphasizing the dependence on feed temperature with reported values ranging from as low as 2.2 kWh/kg-N to an average of 27.2 kWh/kg-N.^241,254^

## 5 Future Nutrient Recovery Technologies

Limitations to conventional technologies such as significant chemical costs, emissions of carbon dioxide, and poor performance at dilute concentrations have limited application for wastewater with low concentrations of nutrients or for wastewater that require excessive pH adjustments. Such shortcomings have motivated interest in biologically assisted precipitation, reverse osmosis, forward osmosis, and electrodialysis for their potential to achieve high nutrient content, eliminate chemical costs, or reduce the emission of carbon dioxide. The following subsections evaluate the fundamental mechanisms, efficiency, and future improvement points of future technologies for effective nutrient recovery.

### 5.1 Biologically Assisted Precipitation of Struvite

Although previous sections focused on struvite precipitation through artificial chemical methods, struvite also occurs naturally as bladder stones, usually coupled with infection from *Proteus* species of bacteria.^255–257^ The biological precipitation of struvite suggests a potential means to increase struvite formation using biomolecules or microorganisms, potentially making struvite formation more economically attractive. Biological methods could also alter the struvite crystal morphology, which affects the rate of nutrient release.^180^ Thus, studies have emerged exploring the concept of using bacteria and biomolecules to enhance struvite precipitation. For example, low-molecular-weight peptides produced by bacteria can promote struvite growth while the metabolism of bacteria can raise the pH and mitigate the need to add a base.^258^ Other peptides like polyaspartic acid, made only of aspartic acid residues, alter struvite crystal morphology, but to the detriment of struvite yield.^259^ A peptide derived from amelogenin proteins, shADP5 (SYENSHSQAINVDRT),^260^ was found to increase the amount of struvite formed and induce changes in crystal morphology that make it more dendritic in nature.^167,261,262^ Specifically, shADP5 tethered to a gold support was included inside the reactor vessel of an electrochemical system, where its presence increased production even at neutral pH.^261^ When shADP5 was expressed on the surface of *E. coli*, it induced more dendritic struvite crystals and higher struvite yield when normalized to the amount of bacteria, which also was found to increase the amount of struvite by more than threefold (Figure 8).^167^ The use of peptides affects the product speciation and morphology by altering crystallization kinetics.^263,264^ Applied for calcium carbonate crystallization, the template effect of peptides resulted in the production of a hollow sphere crystal and was accompanied by a slower transformation process of vaterite to calcite.^263^ Short peptides (introducing a 5–7 residue elongation) affects solubility, and thus, crystallization, depending on the nature and size of the peptide used.^264^ As such, the potential value and scalability of microbial and biomolecular tools for nutrient recovery are significant and are a topic of growing interest.

**Figure 8:**
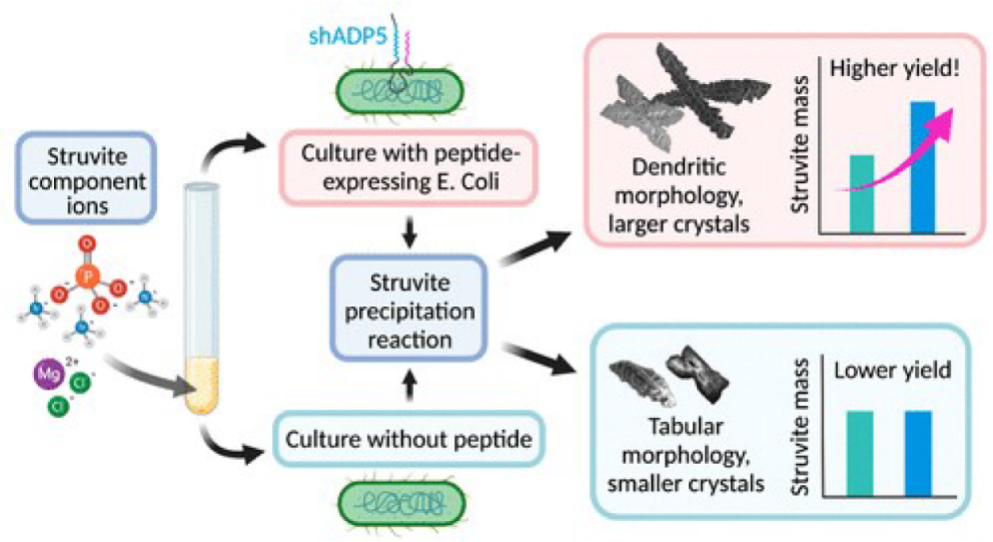
Illustration of a study showing how microbial and biomolecular tools increase struvite yield and modulate struvite morphology.^167^

### 5.2 Membrane-Based Systems

Membrane separation is a physical method that achieves high ammonia recovery efficiencies, low chemical consumption, and simple ammonia recycling.^241^ Currently, membrane separation addresses challenges from conventional methods for ammonia removal and recovery (e.g., air stripping, ion exchange, and biological techniques) such as low removal efficiencies, slow processing rates, and challenges in cost-effective and energetic efficiency.^192^ This section explores the fundamental mechanisms, potential, and challenges of membrane technologies that show potential for nutrient recovery.

#### 5.2.1 Reverse Osmosis and Forward Osmosis

Osmotic pressure is the pressure required to stop water transport across a semipermeable membrane from high water chemical potential (low solute concentration) to low water chemical potential (high solute concentration). This osmotic pressure difference between the two solutions is the driving force for forward osmosis (FO) and reverse osmosis (RO). FO exploits this osmotic pressure to transfer water and solute across the membrane parallel to the concentration gradient. RO artificially applies an equivalent hydrostatic pressure against the concentration gradient and pushes water molecules to the permeate stream.^265^

RO and FO show potential as nutrient recovery technology. Both technologies are well-established for desalination purposes and produce clean water after nutrient recovery. In the case of FO, ammonia removal reaches 66% and phosphate removal 92% when using seawater as the draw stream.^266^ Additionally, sludge dewatering from a secondary wastewater treatment facility achieved 96% NH_4_^+^–N and 98% PO_4_^3–^–P removal.^267^ FO for nutrient recovery purposes in the agricultural field has been implemented at pilot scale and provided fertigation water from WWTP. This system drastically reduced 95% of the organic micropollutants, decreasing bacterial counts and antibiotic resistance genes while adding fertilizer and recovering ammonia and phosphate from WWTP effluent.^268^

For RO membranes that recover ammonia, the retention performance depends strongly on the pH because retention of ammonium is better than that of its deprotonated form (ammonia). RO membranes must have excellent pressure stability to withstand the high operating pressures required, which typically range between 35 and 100 bar.^269^ The pH tolerance of different types of RO membranes varies widely. Thin-film composite (TFC) membranes are usually stable over a wider pH range (2–11) than cellulose acetate (CA) membranes (4–8) and therefore offer greater operational flexibility.^270^ Studies on creating turbulence using glass beads in the form of a fluidized bed over the membrane surface have shown to reduce fouling.^271^ In general, RO achieved maximum concentration factors of 5 at a pressure of 50 bar^272^ with percent nutrient recoveries ranging from 55 to 97% depending on feedstock (urine, manure, and domestic wastewater) with fluxes in the range of 20 L m^–2^ h^–1^ to 25 L m^–2^ h^–1^.^272–274^ An RO pilot-scale study achieved 95% nitrogen removal with a permeate flux of 20 L m^–2^ h^1^ and inlet feedwater pressure of 35 kg cm^2^ from domestic wastewater with tubular membranes and no additional pretreatment.^275^

However, membrane fouling, concentration polarization of solutes near the membrane surface, and regeneration of the draw solution (for FO specifically) significantly inhibit recovery performance of FO and RO.^270^ While RO is affected only by concentration polarization occurring at the external surface of the membrane (ECP), FO is also affected by concentration polarization but occurring on the internal surface (ICP), both of which are problematic for nutrient recovery. ECP reduces the transmembrane pressure, which decreases the water flux, and ICP reduces the concentration difference between the draw and feed solution, resulting in a larger decrease in water flux compared with ECP. This flux reduction ultimately lowers the amount of nutrients recovered. Developing more accurate models for ECP and ICP,^276^ which have recently integrated machine learning approaches,^277^ has broadened understanding and driven new solutions.

To address challenges in fouling and concentration polarization, novel membranes have been explored. Commercially available membranes are often cellulose-based, such as cellulose triacetate (CTA), and polyamide thin-film composition (TFC). TFC is generally more suitable for wastewater purposes because it has better pH stability, higher fluxes and rejection rates, and resistance to biological degradation compared to other cellulose-based membranes.^278,279^ Some strategies to improve membrane performance include 1) tuning the substrate for membranes,^280^ 2) changing the geometry to tubular, spiral, hollow, or flat membranes,^281^ and 3) tuning materials to manipulate membrane properties.^282^ N30F, a pH-stable RO membrane made of poly­(ether sulfone), has been used to recover 10% urea, 55% ammonium, and 94% phosphate from synthetic urine at an operating pressure of 20 bar.^274^ Urine was investigated because urine streams contain up to 80% of the nitrogen products in domestic wastewater.^283^ The study achieved a permeate flux of 20 L m^–2^ h^–1^ and an inlet feedwater pressure of 35 kg cm^–2^. However, spiral-wound membranes used require pretreatment and frequent chemical cleaning, especially for combined domestic-industrial wastewater.^275^

Cleaning methods such as physical cleaning with water or chemical cleaning with acid can help recover flux after fouling from municipal wastewater.^284^ The most energy-consuming step is the draw solution process, which makes the recovery of the solution essential for reducing energy consumption. Some examples of regeneration strategies include nanofiltration, RO, and electrodialysis. Integration of FO with other technologies offers a promising solution by efficiently concentrates the feed stream to levels suitable for downstream processes, achieving ammonia removal of 82%, phosphate removal of 99%, and water recovery of 70%.^285^

#### 5.2.2 Hydrophobic Membrane Contactors

Hydrophobic membrane contactors (MCs) enable the transfer of volatile compounds in the gas phase, such as ammonia, across a membrane interface while preventing liquid water from passing through due to their hydrophobic nature. This characteristic makes them suitable for recovering ammonia from waste streams by allowing selective gas-phase transport, leading to the production of valuable ammonium salt fertilizers.

MC offers several advantages such as a high transfer surface area, relatively low pressure compared to other membrane applications, and negligible contamination in the recovered product.^286,287^ Mass transfer across the hydrophobic membrane is described by the two-film theory while accounting for membrane resistance^288^ (Figure 9). According to the two-film theory, dissolved ammonia (NH_3_(aq)) first diffuses across a concentration boundary layer (δ_L_) from the bulk liquid to the liquid–gas interface at the entrance of the membrane pores. At this interface, NH_3_(aq) volatilizes and ammonia gas (NH_3_(g)) diffuses through the air-filled pores of the hydrophobic membrane. Finally, the ammonia gas dissolves into the acidic receiving solution and reacts instantaneously with protons at the gas–liquid interface to form ammonium NH_4_^+^(aq).^288^ Nitric and phosphoric acids^287,289–291^ can be used as a stripping solution, but sulfuric acid is most commonly used. The driving force for mass transfer in the MC is the ammonia partial pressure gradient between feed and stripping streams^291^ as a result of the concentration difference across the membrane. The ammonia concentration at the gas–liquid interface (acid side) is assumed to be zero because ammonia is instantaneously protonated in contact with the acid stream at low pH.^288^

**Figure 9:**
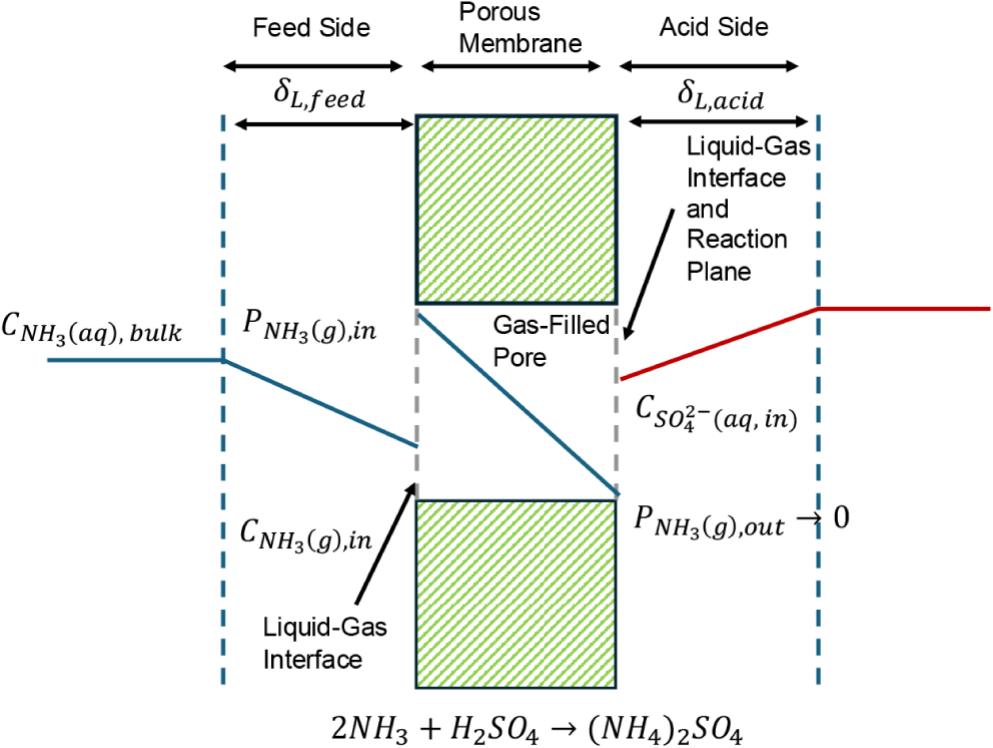
Concentration and partial pressure profiles for ammonia transport. “i″ stands for the gas–liquid interface, and “i in” and “i out” stand for the gas–liquid interface of pore entrance and exit, respectively.

Studies report ammonia recovery between 70 and 100% in MC treating multiple types of wastewater.^288^ Several factors influence ammonia recovery and mass transfer in MC. Increasing the pH of the feed stream enhances both ammonia recovery and the overall mass transfer coefficient, particularly when the pH rises from 8 to 10 or 11.^286,292,293^ However, further increasing the pH beyond 10 or 11 does not provide additional benefits.^286^ The effect of temperature on ammonia removal has also been widely studied^294,295^ with all reports confirming that higher temperatures (between 20 °C and 40 °C) improve ammonia recovery. MC has been investigated for ammonia recovery with feed TAN concentrations ranging from 50 to 3800 mg/L.^288^ Several studies have reported that the initial ammonia concentration does not significantly affect the experimentally determined mass transfer coefficient, as expected since the coefficient inherently normalizes for concentration differences.^292,293^ While some findings suggest that ammonia removal efficiency is also not strongly dependent on the initial concentration,^296^ this may vary depending on system configuration and operating conditions, as higher initial concentrations can increase the driving force for mass transfer and potentially affect removal rates. One operational challenge is the unintended diffusion of water vapor through membrane pores, which can dilute the acid-receiving stream, increase acid consumption, and negatively affect ammonia recovery performance.^288^

Two key degradation mechanisms are fouling and membrane wetting, which can occur independently or in combination. Long-term performance loss due to membrane fouling has received limited attention, mainly because MC remains a relatively new technology with few full-scale applications. Over time, fouling layers can form on the membrane surface, decreasing mass transfer^297^ and causing membrane wetting.^298^ Wetting has been linked to acid leakage into the feed stream in several studies.^298,299^ A reduction in the membrane contact angle after prolonged operation has also been consistently reported.^298^ Implementing appropriate pretreatment to adjust the pH of the feed stream is therefore critical, both for maintaining performance and for controlling process costs. In fact, pH adjustment was identified as the main operational cost in a full-scale MC plant at the Yverdon-les-Bains wastewater treatment facility.^300^ The cost of the TMCS process, including necessary pretreatment but excluding revenue from ammonium sulfate sales, has been reported from various pilot-scale studies. The estimated costs are approximately 3.31 USD/kg-N recovered for rendering condensate wastewater^301^ and 4.87 USD/kg-N recovered for liquid manure.^302^

#### 5.2.3 Electrodialysis

Electrodialysis (ED) is an electrochemical membrane separation technique that utilizes an electric potential to transport salt ions through ion exchange membranes from one solution to another (Figure 10). Ion exchange membranes (IEMs) are composed of a polymer backchain with fixed-charge functional groups and are semipermeable, nonporous, and ionically conductive. IEMs allow the selective passage of counterions (ions with opposite charge of the fixed-charge groups) while excluding passage of coions (ions with the same charge as the fixed-charge groups). This exclusion of coions is called the Donnan effect.^303–305^ The extent of exclusion is determined by the strength of the Donnan potential (eq 5) which arises from the thermodynamic membrane equilibrium, assuming that the membrane is a solution with homogeneously distributed fixed charges.^303^

5 $$\varphi_{\mathrm{Don}}=\varphi^{M}-\varphi^{S}=\frac{RT}{z_{i}F}\ln\frac{a_{i}^{S}}{a_{i}^{M}}$$

**Figure 10:**
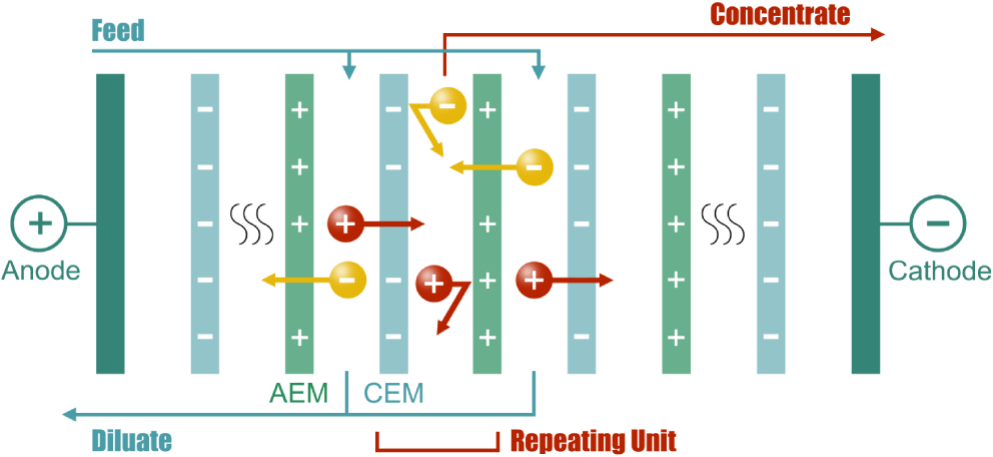
Schematic of standard electrodialysis for the separation of nutrients. Positive charges represent cations, while negative charges represent anions.

In the equation, φ_Don_ is the Donnan potential, φ^M^ and φ^S^ are the membrane and solution potentials, respectively, *R* is the universal gas constant, *T* is the absolute temperature, *z*_*i*_ is the valence of ion *i*, *F* is the Faraday constant, and $a_{i}^{M}$ and $a_{i}^{S}$ are the activities of ion *i* in the membrane and solution, respectively. A larger Donnan potential, thus indicating a greater exclusion of coions, is observed when the fixed charge density increases or concentration of coions in the external solution decreases.^303^

Electrodialysis presents benefits for nutrient recovery such as simultaneous recovery of concentrated nitrogen and phosphorus, production of clean water, elimination of chemical inputs,^306,307^ and potential for integration with sustainable energy sources such as renewable electricity. Performance metrics such as the percent recovery of nutrients, selectivity, and energy consumption are heavily influenced by the nature of the IEMs and the configuration of the electrodialysis cell. Important IEM characteristics include the permselectivity, chemical and mechanical stability, conductivity, and ion exchange capacity. Modifying the degree of polymer cross-linking and the concentration of fixed ion charges was a traditional and straightforward factor for changing the surface modifications of the membrane properties. Recently, novel bulk morphology^308,309^ polymer mixtures (organic–organic and organic–inorganic)^310–312^ and the use of different fixed-charge functional groups in membranes have provided additional tools for engineers to tailor membranes suitable for nutrient recovery.^313,314^

Current electrodialysis literature reports nutrient recovery percentages up to 90% ammonia recovery and 95% phosphate recovery without adding chemicals.^53,315^ Pilot-scale electrodialysis further validated the economics of electrodialysis at larger scales by achieving 4.9 ± 1.5 kWh/kg-N for a product stream of 7100 ± 300 mg NH_4_–N/L and 2490 ± 40 mg K/L (concentration factor of 8) using a 30-cell pair ED cell.^78^

However, electrodialysis suffers from membrane fouling (inorganic and organic), dilution of product due to osmosis, inability to recover high purity nitrogen and phosphorus product streams, and high energy costs when applied for dilute wastewater processing. For example, membrane fouling with scaling caused a decrease in current efficiency to 76 ± 2%,^55,316^ while osmosis could lead to a 400% increase in product volume, ultimately limiting the overall concentration factor.^78^ To address these obstacles, various electrodialysis configurations, in addition to membrane enhancement, were explored in detail for selective nutrient recovery.

Configurations of membranes in electrodialysis systems explored for nutrient recovery include standard electrodialysis of alternating cation and anion exchange membranes, bipolar membrane electrodialysis (BMED),^53,315,317^ selective electrodialysis,^318,319^ and hybrid systems.^55,316,320^ Bipolar membrane electrodialysis implements bipolar membranes to produce acid and base product streams. This pH swing enhances the selective separation of ions by exploiting the thermodynamic equilibrium of ions (e.g., ammonium converts to ammonia under alkaline conditions). Selective electrodialysis uses monovalent membranes to separate counterions based on valency, allowing for separation of monovalent ions (e.g., chloride, nitrate) against di- and trivalent ions (e.g., phosphates).^318^ For example, monovalent anionic membranes were used to distinguish chloride from phosphates and sulfates and monovalent cationic membranes to fractionate divalent magnesium and calcium against ammonium and potassium.^319^ Hybrid systems connect electrodialysis with other unit operations to recover nutrients. Well-known unit operations such as reverse osmosis and air stripping were coupled with electrodialysis to enhance the productivity of ammonium recovery.^55,316^

Future directions for electrodialysis as a nutrient recovery technology include optimizing membranes for selective separation of nitrogen and phosphorus nutrients and integration with pretreatment processes. Ultimately, the performance of all electrodialysis configurations relies heavily on membrane characteristics and thus will require extensive optimization. Investigation of pretreatment process implementation will greatly benefit electrodialysis, as it will maximize electrodialysis performance by mitigating inorganic and organic scaling.

#### 5.2.4 Electrochemical pH-Swing Assisted Electrodialysis

Electrochemical stripping (ECS) has gained attention for highly selective ammonia recovery from wastewater.^321,322^ ECS combines electrodialysis (ED) and membrane stripping to separate ammonia based on charge (using IEMs) and volatility (using gas-permeable membrane, GPM).^323,324^ This hybrid system enhances the selective concentration of ammonia from the ED concentrate. Compared to conventional ammonia stripping that uses lime or caustic to convert aqueous NH_4_^+^ to volatile NH_3_,^325^ ECS electrochemically introduces a pH swing that separates ammonia from other components of wastewater.

The fundamental mechanism of ECS involves utilizing an electric driving force to transport cations from one compartment to another in an electrochemical cell, thus producing an ammonium-concentrated stream. The pH of the concentrate chamber is then increased electrochemically beyond the p*K*_a_ of NH_4_^+^/NH_3_ system (=9.25), facilitating the conversion of ionized ammonium (NH_4_^+^) to ammonia (NH_3_). The relative distribution of the two forms in a given aqueous solution is described by the following equation:^326^

6 $$\alpha \left(t\right)=\frac{c_{{\mathrm{NH}}_{3}}\left(t\right)}{c_{\mathrm{TAN}}\left(t\right)}=\frac{K_{a}}{K_{a}+10^{-\mathrm{pH}(t)}}$$

where α is the ratio of the concentration of ammonia over total ammonia nitrogen (NH_4_^+^ + NH_3_) and *K*_a_ is the dissociation equilibrium constant. The elevated pH causes NH_4_^+^ to deprotonate NH_3_. This basic stream containing NH_3_ is then put in contact with an acidic stream through a hydrophobic gas-permeable membrane (Figure 11). The transmembrane difference in ammonia vapor pressure between the two streams causes the volatile ammonia to diffuse through the membrane to the acidic trap chamber for collection. Other nonvolatile components cannot permeate across the membrane and thus remain in the basified stream.^326,327^ The ammonia vapor flux through the gas-permeable membrane is expressed by the following equation:^326^

7 $$J_{{\mathrm{NH}}_{3}}=L_{p}\cdot k_{H}\left(c_{{\mathrm{NH}}_{3}}^{b}-c_{{\mathrm{NH}}_{3}}^{a}\right)$$

where *L*_p_ is the ammonia permeability across the gas-permeable membrane, $c_{{\mathrm{NH}}_{3}}^{b}$ and $c_{{\mathrm{NH}}_{3}}^{a}$ are the ammonia concentrations in the basified stream and stripping acid streams, respectively, and *k*_H_ is Henry’s constant of ammonia.

**Figure 11:**
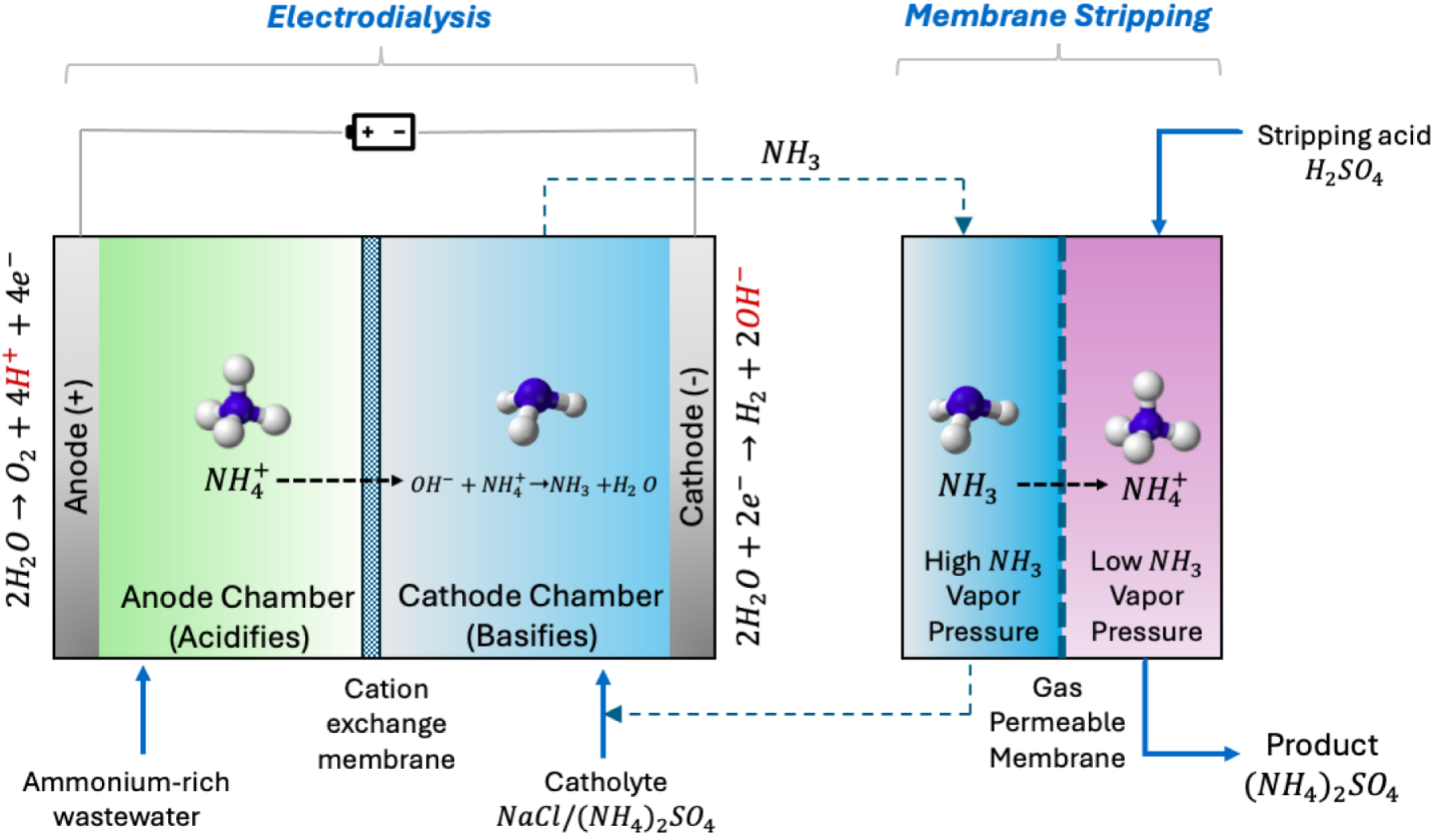
Representative schematic of an electrochemical stripping (electrodialysis + membrane stripping) system for ammonia recovery.

Researchers have investigated three methods for achieving an electrochemically induced pH swing, including 1) water splitting reactions at the electrodes,^328,329^ 2) bipolar membranes (BPM),^326,327,330^ and 3) proton-mediated redox couple reactions.^331^ Experiments using a three-chambered parallel plate reactor with a cation exchange membrane (CEM) between the anode and cathode chambers and a GPM between the cathode and trap chamber demonstrated ammonia recovery efficiency of 93% in batch experiments at a current density of 100 A m^–2^ with real urine (3,820 mg NH_4_^+^/L).^328^ This system required 30.6 MJ kg^–1^ N in continuous-flow experiments, which is slightly less than the conventional ammonia stripping. Hydrogen evolution reaction in the cathode chamber produced an alkaline environment that helped achieve the desired pH swing, allowing ammonia to diffuse across the gas-permeable membrane into the trap chamber. Another study investigated cathodic feeding vs anodic feeding of a synthetic solution containing 3,608 mg NH_4_^+^/L and reported higher ammonia recovery for cathodic feeding (93.2% vs 79.1%) in a similarly configured system.^329^ This study also reported improved ammonia recovery with the use of Na_2_HPO_4_ as an anolyte compared to Na_2_SO_4_. This was attributed to the rapid pH increase in the anode compartment due to pH buffering effect of HPO_4_^2–^, which enhanced the conversion of NH_4_^+^ to NH_3_.^332^

Bipolar membranes have also been used to electrically drive water dissociation to increase the pH of the feed stream while simultaneously producing an acid stream for ammonia stripping in a membrane contactor.^326^ This configuration does not require a continuous external acid or buffer input to strip ammonia from the ED concentrate as the stripping acid was produced within the system itself, resulting in 67.5% recovery of ammonia at a current density of 200 A m^–2^ and energy consumption of 25.8 MJ kg^–1^ N for a synthetic source-separated urine (3772 mg NH_4_^+^/L). The study also demonstrated that increasing the critical pH beyond 11 results in only marginal improvement of ammonia recovery at the expense of greater energy consumption.

A number of researchers have investigated nitrogen removal by adsorption of NH_4_^+^ from feed wastewater onto solid ion exchange resins. Weak acid cation exchange resins (WACs) have proven to have high capacity for ammonium-nitrogen removal from real urine.^223^ However, WACs require chemically intensive regeneration that makes them energetically unfavorable. To overcome this barrier, WACs have been used to concentrate ammonia from synthetic urine and BPM used to generate protons for WAC regeneration.^330^ Membrane stripping has been subsequently used to recover the eluted ammonium from the WAC. Though this configuration reduces the material cost for resin regeneration, it required a continuous input of sulfuric acid to recover the ammonia. Another study used a four-compartment ED cell comprising anion exchange membrane (AEM), BPM, and CEM to produce two acid streams through oxygen evolution reaction (OER) at the anode and bipolar membrane.^327^ The acid stream produced in the anode compartment through the OER was used to regenerate the ammonium-laden ion-exchange resin used for concentrating synthetic wastewater. The other acid stream produced from proton generation by BPM was used to strip the ammonia in a membrane contactor system. This system significantly reduced external chemical inputs and achieved ammonia recovery efficiency of 9–74% at current densities of 5–25 A m^–2^ while consuming 29.2–42.8 MJ kg^–1^ N of energy for synthetic domestic wastewater containing 50 mg NH_4_^+^/L.

Proton-mediated electrochemical reactions are an alternative to the high voltage (0.83 V for BPM, 1.23 V for water electrolysis) and membrane costs associated with BPM applications for large-scale.^333^ For example, electrochemical oxidation and reduction of hydroquinone (HQ) and benzoquinone (BQ) increased the solution pH in the cathode compartment due to H^+^ consumption and decreased in the anode due to H^+^ release, reaching ∼50% recovery of NH_4_ +^331^ at an energy consumption of 11.52 MJ kg^–1^ N for a synthetic wastewater containing 90 mg NH_4_^+^/L. Higher solution pH aided in improved flux and recovery; however, the use of HQ/BQ produced a maximum achievable pH of 10, which is relatively low compared to ED or BPM-ED limiting ammonia recovery efficiency.

In order to scale up electrochemical pH-swing ammonia recovery, future systems will require the integration of electrodialysis with large stacks of ion exchange membranes and hollow fiber membrane contactors (HFMCs) capable of processing high wastewater volumes. This coupling has been shown to achieve high ammonia concentration factors, approaching commercial grade levels of ∼10 wt % N at energy consumption of 6.8–22.1 MJ/kg N for synthetic CAFO wastewater with 524 mg NH_4_^+^/L.^334^ The use of multistack ED configurations allows for effective preconcentration of ammonium, while the HFMC selectively recovers the concentrated ammonia in volatile form, together providing a scalable and energy-efficient pathway for nitrogen recovery from wastewater.

Ammonia recovery using an electrochemically induced pH swing has been successfully studied at lab scale, but few pilot studies have been conducted. An onsite pilot-scale BPM-ED coupled to membrane stripping including 65 cell pairs and 3.15 m^2^ membrane area has been used to treat 150 L of anaerobic digester centrate (485 mg NH_4_^+^/L) per hour to produce a concentrated ammonium sulfate product.^335^ The pilot plant recovered 235.7 g-Nd^1–^, while consuming 22.68 MJ kg^–1^ N. Scaling and fouling limited the operation of the membrane stack, and daily cleaning procedures interrupted continuous operation. The same pilot plant was used in another study to treat source-separated diluted urine (1 g-NH_4_^+^/L).^336^ Unlike laboratory-scale studies, influent nitrogen loading and current density did not directly influence ammonia removal, while the effluent pH was found to be directly related to the ammonia removal efficiency. The pilot plant achieved 88% recovery of ammonia from urine at a current density of 50–200 A m^–2^ while consuming 46.8 MJ kg^–1^ N of energy when the effluent pH was around 4. Although high ammonia recovery was achieved, this system suffered from high overall membrane/compartment resistance due to the large number of stacked cell pairs leading to increased resistance compared to lab-scale ED scenarios.^337^ Additionally, the formation of salt bridges across the inlet and outlet manifolds allowed ions to move through the liquid rather than cross the ion exchange membranes, impacting nitrogen removal efficiency and energy consumption.

ECS presents a promising pathway for energy-efficient and selective ammonia recovery, especially when integrated with electrodialysis and gas-permeable membranes. Literature consistently demonstrates high ammonia recovery efficiencies (>90%) under optimized pH and current densities, with energy demands often lower than conventional stripping. However, energy consumption varies widely (7–47 MJ/kg-N), driven by system design, feed composition, and pH control strategy. Notably, BPMs offer self-sustained acid/base generation but suffer from high voltage and cost, while redox-mediated systems reduce voltage but limit achievable pH. Trade-offs between recovery efficiency, system complexity, and chemical input requirements suggest that hybrid designs combining ion-exchange concentration, electrochemical pH-swing, and HFMC stripping—may provide the most scalable and sustainable configurations. Future research should focus on minimizing internal resistance, controlling ion shortcut pathways, and extending pilot-scale validation under realistic wastewater conditions to support broader implementation.

## 6 Comparison of Emerging Nutrient Recovery Technologies

Here, we present a comparative analysis of common nutrient separation technologies introduced throughout this review (Tables 5–7). The results will inform future researchers and engineers of both the qualitative benefits and the weaknesses of each technology and provide a quantitative comparison of performance based on crucial nutrient recovery operation metrics. All nutrient recovery technologies listed in Tables 5–7 have focused on nitrogen recovery with only struvite precipitation harnessing phosphorus nutrients.

**Table 5:** Comparison of Performance Metrics for State-of-the-Art Nutrient Recovery Technologies^a^

Technology	P-content achieved (%)	N-content achieved (%)	Percent Recovery (%)	Energy (MWh/ton-N)	Chem. Cost ($/ton-N)	Op. Cost ($/ton-N)	Conc. Factor (−)
**Conventional Technologies**							
Ammonia Stripping^340–343^	0	6.4–8.1	80–95	1.2–4.0	1,900–3,000	2,000–3,200	38–55
Struvite Precipitation^340,343,344^	12.6	5.7	74–99	0.6–1.3	11,100–14,500	11,100–14,600	38–49
Ion Exchange^340,343,345^	0	0.1	77–95	0.1	200–7,400	200–7,400	14–28
Membrane Distillation^242–340,340–349^	0	6.2	90–98	0.2–1.3	0–1,400	100–1,400	5–6
**Future Technologies**							
Electrodialysis^78,316,350–352^	0	0.9–2.7	62–95	3.7–4.9	0	200–300	3–9
Membrane Contactor^195,353–355^	0	0.5–1.5	87–98	0.1–0.2	1,400–1,500	1,400–1,500	3–8
Reverse Osmosis^340,343,356–358^	0	0.5	70–94	3.7–4.7	600–700	800–1,000	4–5
Electrochemical Stripping^326,328–330,334^	0	1.9–14.5	50–93	3.2–13	0–1,400	300–700	3–200

a Operating cost incorporates energy and chemical cost.

**6:** Summary and Future Directions of State-of-the-Art Nutrient Recovery Technologies^b^

Technology	TRL^a^	Advantages	Disadvantages	Gap Analysis	Optimal Feedstock^a^
**Conventional Technologies**					
Ammonia Stripping	High	• Straightforward operation design	• CO_2_ emissions	• Minimizing chemical cost	• WWTP-centrate
		• Cost effectiveness	• Chemical costs	• Utilization of waste heat	• CAFO waste
		• Design optimizations			
Struvite Precipitation	High	• Simultaneous N and P recovery	• Chemical additives	• Understanding of competing chemistry in WW matrices	• WWTP-centrate
		• Solid fertilizer product	• Operation costs	• Enhanced crystal management	• WWTP-primary sludge liquid
			• Byproducts diminish productivity		• Fertilizer production effluent
					• CAFO waste
Ion Exchange	High	• Application for both N and P recovery	• Low N wt % product	• Investigation of adsorbent materials for long-term sorption	• WWTP-centrate
		• Low energy consumption	• Chemicals for adsorbent regeneration		• Fertilizer product effluent
					• CAFO waste
					• WWTP-Secondary treatment effluent
					• WWTP-discharge
Membrane Distillation	High	• Clean water production	• Membrane wetting and fouling	• Operation condition optimization	• WWTP-centrate
		• High N wt % product	• Thermal energy required	• Membrane materials	• CAFO waste

a Low TRL (1–5): laboratory studies performed, Medium TRL (6–7): pilot-scale studies performed, High TRL (8–9): full-scale studies performed.^16^

b Assuming preliminary treatment of large suspended solids using screens, grinders, or grit chambers.

**Table 7:** Summary and Future Directions of State-of-the-Art Nutrient Recovery Technologies (Continued)

Technology	TRL^a^	Advantages	Disadvantages	Gap Analysis	Optimal Feedstock^b^
**Future Technologies**					
Electrodialysis	Medium	• No chemical inputs	• Membrane fouling	• Nutrient selective membranes	• WWTP-centrate
		• Simultaneous recovery of N and P	• Dilution of product due to osmosis	• Integration with pretreatment processes	• CAFO waste
			• High energy costs at dilute concentrations		
Membrane Contactor	Medium	• High transfer surface area	• Membrane fouling	• Low-cost pH adjustment needed	Digestate from AD plant
		• Low pressure operation	• High chemical costs	• Membrane design to reduce unintended water transport	• CAFO waste
					• WWTP centrate
Reverse and Forward Osmosis	Medium	• Clean water production	• Lack of selectivity	• Membrane materials	• WWTP-secondary treatment effluent
		• Well-established for desalination	• Membrane fouling	• Optimization of draw solution regeneration	• WWTP-discharge
			• Concentration polarization	• Hybrid approach to increase concentration	• Landfill leachate
Electrochemical Stripping	Low	• High N wt % product	• Membrane fouling	• Operating condition and cell configuration optimization	• WWTP-centrate
		• High concentration factor	• High energy costs at dilute concentrations	• Membrane fouling	• WWTP-primary clarifier effluent
				• Scale-up	• CAFO waste
					• Landfill leachate

a Low TRL (1–5): laboratory studies performed, Medium TRL (6–7): pilot-scale studies performed, high TRL (8–9): full-scale studies performed.^16^

b Assuming preliminary treatment of large suspended solids using screens, grinders, or grit chambers.

In general, conventional technologies have reported high concentration factors in the range 5–55 with high nitrogen contents of up to 8.1%. Future technologies introduced in this review report concentration factors of 3–9 with 0.5–2.7 N wt %. This discrepancy largely stems from the fact that conventional technologies have been employed at large scale for decades while future technologies such as electrodialysis and reverse osmosis have only recently been tested for nutrient recovery purposes.^78,158^ However, electrochemical stripping shows a high potential for reaching fertilizer-level concentrations. Coupling electrodialysis with hydrophobic hollow-fiber membrane contactors produced a 14.5 N wt % stream and concentration factors of 200.^334^ This performance underscores how integrated state-of-the-art technologies can create synergetic effects that deliver an above-average efficiency. For example, integration of ED and struvite precipitation can recover both nitrogen (concentrate) and phosphorus (struvite), diversifying the final products.^338^ Membrane distillation paired with nitrogen-selective ion exchange adsorbents benefits both processes by introducing selective separation of nutrients, achieving high concentration factors, and reducing membrane fouling.^339^ Thus, future integration of multiple technologies will be crucial to complement fundamental limitations of each technology and reach fertilizer-level concentrations (200–320 g-N/L) using dilute wastewater (0.001–1 g-N/L).

High chemical costs are a persistent problem for many recovery technologies, increasing the total operating costs. For example, struvite precipitation requires $11,000–14,600/ton-N for magnesium and alkali chemicals to provide magnesium and foster optimal pH levels to maximize struvite precipitation. Large-scale struvite precipitation introduces low-grade magnesium or integrates with other unit operations such as ion exchange, mitigating chemical costs, but still chemicals contribute to 77–99% of the total operating costs.^173^ Ion exchange incurs $200–7,400/ton-N, with the higher range primarily due to the price of zeolite and regenerant brine consumption. However, these costs could potentially be reduced by optimizing the number of regeneration cycles and exploring different regenerants, which are crucial for supplying Na^+^ cations to zeolite materials. Contrary to ion exchange and struvite precipitation, electrodialysis and membrane distillation eliminate or minimize addition of chemicals, which significantly decreases operating costs and ensures chemical safety. Specifically, bipolar membrane electrodialysis produces base and acid from wastewater, presenting promise as a supplementary technology for crystallization, ion exchange, and ammonia stripping to eliminate external chemical additives.^315,317^

Integration of recovery technologies with existing wastewater infrastructure is crucial to minimize the upfront capital cost. Optimal feedstock for recovery technologies was a centrate stream in WWTPs or CAFO waste streams. This stems from the fact that many technologies require moderate to high-concentration feed streams for efficient nutrient recovery, typically over 100 mg-N or P/L.^190,359^ Struvite precipitation could also be used with fertilizer production effluent and liquid from the primary sludge in WWTP because it requires lower levels of nutrients, as long as both ammonium and phosphorus are present. Ion exchange and adsorbents, reverse osmosis, and forward osmosis provide additional value propositions of complying with discharge regulations when added downstream of municipal WWTP. However, there is a significant gap in achieving efficient separation at dilute concentrations at large volumes, and these must be considered when developing technologies.

Although this comprehensive comparison of state-of-the-art technologies serves as a valuable reference for researchers involved in nutrient recovery, additional metrics such as capital costs, maintenance costs, and labor costs should be considered in the context of full-scale plants. A detailed comparison based on technoeconomic analysis and life cycle assessment would be beneficial for full-scale implementation. Nonetheless, conventional technologies such as ammonia stripping, struvite precipitation, and ion exchange can be immediately implemented with various wastewater feedstock such as WWTP centrate, CAFO waste, and WWTP primary sludge liquid identified in Sections 2.1 and 3.1. This insight has already been verified by the successful operation of various large-scale nutrient recovery installations. However, robust integration of spatiotemporal monitoring of wastewater nutrients with technology operation must be pursued to reach commercialization.^153,154^ Reliable product quality and the amount of chemical addition are crucial factors for determining optimal operating conditions and product economics for companies. Enhancement in purity and reliability of recovered products through dynamic adjustments of operating conditions should be evaluated to improve the commercial viability of full-scale installations. In order to overcome the significant chemical and overall costs and achieve higher concentration products, integration of future technologies such as electrodialysis, bioadditives, and hydrophobic membrane contactors is necessary. Integrating conventional and emerging technologies could also diversify recoverable products and improve nutrient recovery productivity. For example, struvite precipitation introduces a solid fertilizer product, while reverse osmosis and ion exchange provide clean water. Sequential integration of bipolar membrane electrodialysis, struvite crystallization, and membrane capacitive deionization recovers nutrients in the form of both struvite and aqueous nitrogen-rich streams, achieving approximately 100% PO_4_^3–^–P and 77% NH_4_^+^–N recovery.^360^

Acknowledging this clear path for technology integration, investigation of performance enhancements or disruptions when coupling different technologies is essential. For example, the integration of liquid–liquid membrane contactors and electrodialysis achieved a high maximum nitrogen product of 15 N % but resulted in varying concentration factors dependent on the form of ammonium salt recovered from the membrane contactor stage.^361^ Membrane distillation crystallization (membrane distillation followed by crystallization) can recover both nutrient salts and clean water with relatively lower pressure and temperatures but introduce the risk of crystallization in the module and the existence of concentration and temperature polarizations.^362^ Understanding comprehensive systems of different technologies will inform the community of optimal technology systems for specific wastewater streams. In addition, future research should explore the compatibility of not only influent and effluent of wastewater sources with recovery technologies but also specific streams in WWTPs and effluents from other unit operations. Feedstock pH, coexisting compounds (heavy metals, organic compounds, and solid substances), and spatiotemporal variations of wastewater affect recovery performance and product composition from technologies. Specifically, the operation concentration window, economics, and concentration factor must be explored to streamline integration of technologies to specific wastewater streams or other recovery technologies. These blocks of information will ultimately address obstacles toward nutrient recovery commercialization and contribute to constructing a decision tree based on optimal nutrient recovery technologies for the wastewater to be treated.

## 7 Conclusions and Perspectives

With an increasing demand for a circular fertilizer manufacturing process, the implementation of nitrogen- and phosphorus-based nutrient recovery systems is crucial. Significant opportunities remain in harvesting trillions of gallons of waste streams rich in nitrogen and phosphorus nutrients. Specifically, nutrient recovery from municipal wastewater, CAFO waste, and landfill leachate shows promise to offset 5 million tons of nitrogen and 0.4 million tons of phosphorus from global fertilizer demand, with advanced spatiotemporal approaches capturing the dynamic variations of wastewater compositions. Despite the existence of nutrient recovery companies, the predominant methods of managing nitrogen and phosphorus remain focused on the removal and direct application of wastewater to crops. This underutilization of nutrient recovery technologies, along with the sheer volume and concentration of waste streams, demonstrates the need to further develop viable, burgeoning nutrient recovery methods.

This review examined the current and feasible near-future opportunities for nutrient recovery technologies in the circular fertilizer process. Established technologies, such as ammonia stripping, ion exchange, membrane distillation, and struvite precipitation, offer practically applicable strategies demonstrating high-concentration factors, high percent recovery, and moderate energy consumption. Optimization in reactor design and materials along with a thorough understanding of fundamental mechanisms have substantially contributed to the high-performance metrics. However, high chemical and operation costs are critical barriers for optimization. Near-future opportunities, such as electrodialysis, membrane contactors, reverse and forward osmosis, and electrochemical stripping, mitigate these limitations by eliminating chemical addition, achieving high concentration factors, and enabling the use of renewable electricity. However, design and material optimization, energy consumption reduction, and selectivity enhancement are future research gaps to be addressed for full-scale installments of future technologies. In parallel with individual technological advancements, the integration of distinct technologies must be investigated. The distinct, complex, and dynamic nature of wastewater and performance enhancements and obstacles unique to process integration necessitate a thorough investigation of holistic nutrient recovery systems to fully harness the nutrient potential across diverse wastewater streams. Beyond individual technological advancements, specific future steps include integration of spatiotemporal monitoring of feedstock with process operating conditions, compatibility testing of technologies with WWTP streams and effluents from unit operations, and investigation of performance enhancements from technology integrations. Ultimately, this will contribute to the realization of a sustainable nutrient cycle.

## Supplementary Material



## Notes

Compiled data of nutrient availability in different wastewater sources (PDF)
