# Supplementary material for: Nutrient Separation Systems: Current Progress and Future Opportunities
Source: ACS ES T Eng. 2026 Jan 20;6(2):567–96. doi: 10.1021/acsestengg.5c00743 (PMC12910600; doi:10.1021/acsestengg.5c00743)
Supplement: Supplementary file 1 [file ee5c00743_si_001.pdf]

# Supporting Information - Nutrient Separations Systems: Current Progress and Future Opportunities

Hyuck Joo Choi[1], Mohammed Tahmid[1], Luisa Barrera[2], Christian E. Alvarez-Pugliese[3], Danae A. Chipoco Haro[4], Dylan J. Weber[1], Wilfredo J. Cardona-Velez[3], Bengu Mete[3], Dayana Donneys Victoria[3], Zhengwen Zhang[5], Victor K. Lim[6], Olatunde Akanbi[7], Jacob D. Hostert[8], Archer Montgomery[3], Divya Ganesan[1], Erika I. Barcelos[7], Jie Xu[6], Joseph K. Scott[1], Gerardine G. Botte[3], Kayleigh Millerick[9], Chris Yuan[5], Julie N. Renner[8], Roger H. French[7], Marta C. Hatzell[*,2]

[1]School of Chemical and Biomolecular Engineering, Georgia Institute of Technology, Atlanta, GA 30309, USA;

[2]George W. Woodruff School of Mechanical Engineering, Georgia Institute of Technology, Atlanta, GA 30309, USA;

[3]Chemical Electrochemical Technology Innovation Lab, Institute for Sustainability and Circular Economy, Dept. of Chemical Engineering, Texas Tech University, Lubbock, TX 79409, USA;

[4]School of Materials Science Engineering, Georgia Institute of Technology, Atlanta, GA 30332, USA;

[5]Dept. of Mechanical Aerospace Engineering, Case Western Reserve University, Cleveland, OH 44106, USA;

[6]Intelligent Sustainable Technologies Division, Georgia Institute of Technology, Atlanta, GA 30318, USA;

[7]Dept. of Materials Science Engineering, Case Western Reserve University, Cleveland, OH 44106, USA;

[8]Dept. of Chemical Engineering, Case Western Reserve University, Cleveland, OH 44106, USA;

[9]Dept. of Civil, Environmental, Construction Engineering, Texas Tech University, Lubbock, TX 79409, USA

*Email: marta.hatzell@me.gatech.edu

Table 1: Annual total nitrogen and phosphorus effluent loads and reported concentrations by industrial point-source category.[1]

| **Industrial Point Source Category** | **Annual Total N Load** (million lbs) | **Annual Total P Load** (million lbs) | **Reported N Conc.** (mg-N/L) | **Reported P Conc.** (mg-P/L) |
|---|---|---|---|---|
| Construction and development | 71.4 | 14.5 | 3.27 | 0.550 |
| Hospital | 66.1 | 24.0 | 1.96 | 1.47 |
| Pulp, paper and paperboard | 44.9 | 7.3 | 1.58 | 0.329 |
| Gum and wood chemicals manufacturing | 42.8 | 2.8 | 0.175 | 0.73 |
| Meat and poultry products | 39.2 | 42.2 | 14.7 | 1.18 |
| Drinking water treatment | 31.3 | 1.3 | 0.113 | 0.33 |
| Steam electric power generating | 30.1 | 3.18 | 0.0945 | 0.0371 |
| Explosives manufacturing | 18.7 | 0.17 | 6.34 | 0.0802 |
| Organic chemicals, plastics and synthetic fibers | 14.1 | 5.41 | 0.523 | 0.164 |
| Metal finishing | 10.6 | 3.44 | 0.264 | 0.0706 |
| Iron and steel manufacturing | 4.7 | 19.6 | 0.251 | 0.0708 |
| Mineral mining and processing | 3.2 | 7.88 | 0.566 | 0.0471 |
| Plastics molding and forming | 6.3 | 7.28 | 0.273 | 0.333 |

*Reported Conc. is the median (50th percentile) from each category in 2018.*

# References

(1) EPA's Review of Nutrients in Industrial Wastewater Discharge. 2020; https://downloads.regulations.gov/EPA-HQ-OW-2018-0618-0659/content.pdf.
